# Role of body mass index and weight change in the risk of cancer: A systematic review and meta-analysis of 66 cohort studies

**DOI:** 10.7189/jogh.14.04067

**Published:** 2024-03-29

**Authors:** Xiaoye Shi, Gengwen Deng, Haiteng Wen, Anqi Lin, Haitao Wang, Lingxuan Zhu, Weiming Mou, Zaoqu Liu, Xiaohua Li, Jian Zhang, Quan Cheng, Peng Luo

**Affiliations:** 1 Department of Oncology, Zhujiang Hospital, Southern Medical University, Guangzhou, Guangdong, China; 2 The Second School of Clinical Medicine, Southern Medical University, Guangzhou, Guangdong, China; 3 The First School of Clinical Medicine, Southern Medical University, Guangzhou, Guangdong, China; 4 Thoracic Surgery Branch, Centre for Cancer Research, National Institutes of Health, Bethesda, Maryland, USA; 5 Department of Aetiology and Carcinogenesis, National Cancer Centre, National Clinical Research Centre for Cancer, Cancer Hospital, Changping Laboratory, Chinese Academy of Medical Sciences and Peking Union Medical College, Beijing, China; 6 Department of Urology, Shanghai General Hospital, Shanghai Jiao Tong University School of Medicine, Shanghai, China; 7 Key Laboratory of Proteomics, Beijing Proteome Research Centre, National Centre for Protein Sciences, Beijing Institute of Lifeomics, Beijing, China; 8 Key Laboratory of Medical Molecular Biology, Institute of Basic Medical Sciences, Chinese Academy of Medical Sciences, Department of Pathophysiology, Peking Union Medical College, Beijing, China; 9 Department of Respiratory and Critical Care Medicine, Sixth People’s Hospital of Chengdu, Chengdu, Sichuan, China; 10 Department of Neurosurgery, Xiangya Hospital, Central South University, Changsha, Hunan, China; 11 National Clinical Research Centre for Geriatric Disorders, Xiangya Hospital, Central South University, Hunan, China

## Abstract

**Background:**

This study was designed to evaluate the effects of body mass index (BMI) and weight change on the risk of developing cancer overall and cancer at different sites.

**Methods:**

We searched PubMed and other databases up to July 2023 using the keywords related to ‘risk’, ‘cancer’, ‘weight’, ‘overweight’, and ‘obesity’. We identified eligible studies, and the inclusion criteria encompassed cohort studies in English that focused on cancer diagnosis and included BMI or weight change as an exposure factor. Multiple authors performed data extraction and quality assessment, and statistical analyses were carried out using RevMan and *R* software. We used random- or fixed-effects models to calculate the pooled relative risk (RR) or hazard ratio along with 95% confidence intervals (CIs). We used the Newcastle-Ottawa Scale to assess study quality.

**Results:**

Analysis included 66 cohort studies. Compared to underweight or normal weight, overweight or obesity was associated with an increased risk of endometrial cancer, kidney cancer, and liver cancer but a decreased risk of prostate cancer and lung cancer. Being underweight was associated with an increased risk of gastric cancer and lung cancer but not that of postmenopausal breast cancer or female reproductive cancer. In addition, weight loss of more than five kg was protective against overall cancer risk.

**Conclusions:**

Overweight and obesity increase the risk of most cancers, and weight loss of >5 kg reduces overall cancer risk. These findings provide insights for cancer prevention and help to elucidate the mechanisms underlying cancer development.

**Registration:**

Reviewregistry1786.

Cancer is a major global health issue and one of the leading causes of disease-associated mortality [1]. Approximately 19–20 million people worldwide are diagnosed with cancer annually, while approximately 10 million people die from cancer [2]. The five-year survival rate for some malignancies, such as hepatocellular carcinoma, is less than 15%, whereas that for lung cancer is approximately 4–17% [3,4]. Various risk factors for cancer development have been identified, including dietary habits, alcohol consumption, smoking, metabolic syndrome, and diabetes [5–9]. In recent years, research has also indicated a potential link between overweight or obesity and weight change and the risk of certain cancers [10–15].

To date, the effect of overweight or obesity on cancer risk has not been thoroughly studied, and there are controversies among the findings of many studies. While some studies have shown that obesity increases the risk of gastric cancer [16,17], other studies have shown no significant association [18–22]. These discrepancies in findings may be due to inconsistencies in study type (cohort or case-control), differing definitions of obesity, ethnic differences, or sample size. Furthermore, existing meta-analyses have focused mainly on the effect of overweight or obesity on the risk of specific cancer [11,13,23–26]. Limited attention was given to the impact of underweight on cancer risk or the effect of different types of body mass index (BMI) on the risk of cancers in different systems.

Moreover, the current research on the relationship between weight change and cancer risk is also subject to controversy and limitations. For instance, one study concluded that weight gain increases the risk of breast cancer [27], yet another study suggested that the effect of weight gain on breast cancer varies depending on different oestrogen and progesterone receptor statuses [28]. Furthermore, most meta-analyses examining the impact of weight loss on cancer risk have focused on intentional weight loss rather than unintentional [12,29]. These factors may have affected their findings. Moreover, few meta-analyses assessing the effect of weight change on cancer risk have distinguished between different degrees of weight change.

Through a meta-analysis of cohort studies, we explored the risk of cancer in populations with different abnormal BMIs (including underweight, overweight, and obese individuals) compared to that in individuals with a normal BMI, as well as the risk of cancer in populations with different weight changes compared to those with unchanged weight. We specifically included cohort studies and set consistent cutoff values for the BMI categories in the meta-analyses to minimise bias. In addition, we examined the differences in cancer risk according to BMI category or weight change for patients with different cancers and attempted to explain these differences through cancer-related mechanisms. This may have important implications for cancer prevention and further exploration of the mechanisms underlying the relationship between BMI or weight change and cancer risk.

## METHODS

We conducted this systematic review and meta-analysis according to the guidelines of the Preferred Reporting Items for Systematic Reviews and Meta-analysis (PRISMA) 2009 statement (Table S1 in the **Online Supplementary Document**) [30]. The protocol for this systematic review and meta-analysis was registered on the online registration platform Researchregistry. The study was assigned a unique identification number (Reviewregistry 1786), and can be found by searching the registry’s database of registered systematic reviews and meta-analyses using this number.

### Search strategy

The literature search included the search of PubMed, Web of Science, Medline, Scopus, Cochrane, EconLit, Embase, Food Sciences and Technology Abstracts, and PsycINFO databases up to July 2023. The search string was (risk) AND (cancer OR carcinoma) AND (weight OR overweight OR obesity) (Table S2 in the **Online Supplementary Document**).

### Eligibility criteria

Three authors checked all retrieved studies for eligibility; when there was a disagreement, the fourth and fifth authors joined the discussion to determine the final results. We included studies in the meta-analysis if they met the following criteria: involved a cohort study; involved a cancer diagnosis; indicated a BMI or weight change; had a reported headcount ratio or unadjusted relative risk (RR) or hazard ratio (HR); and were published in English. We excluded studies if the full text was not available, relevant data were not reported or were unavailable, or the study was a duplicate.

### Data collection process

Two authors extracted the data. The team members independently checked and then cross-checked the data to achieve consistent results. The following variables were recorded for each study: first author’s name, year of publication, sample size, sex and age of the baseline population, BMI classification criteria or mode of weight intervention and degree of weight change, raw headcount ratio data or unadjusted RRs or HRs with their 95% confidence intervals (CIs).

### Exposure definition

For the classification of BMI, we used the weight classification prescribed by the World Health Organization (WHO). BMI = weight (kg)/height (m^2^); underweight (BMI<18.5), normal weight (BMI = 18.5–25), overweight (BMI = 25–30), and obesity (BMI≥30) [31]. We also defined two categories – underweight or normal (BMI<25) and overweight or obesity (BMI≥25).

For the classification of weight change, we grouped the data on weight gain and weight loss provided by the included studies into groups: weight gain >2 kg, weight gain >5 kg, weight gain >10 kg, weight gain >20 kg, weight loss >2 kg and weight loss >5 kg. No weight change was defined as weight gain or weight loss not greater than the threshold value set for each subgroup.

### Statistical analysis

We determined the heterogeneity between studies by the χ^2^ test and the *I^2^* index. Both random- and fixed-effects models were used to estimate the combined effects. When substantial heterogeneity was observed (*I^2^*>50%), we chose the random-effects model to effectively address the heterogeneity in the meta-analysis. This model accommodates varying true effect sizes between studies, incorporating this variability into the analysis. Conversely, when heterogeneity was minimal (*I^2^*≤50%), we utilised the fixed-effects model, assuming a single true effect size across all studies. We assessed the potential publication bias by funnel plots and Egger’s test [32]. A result with a two-tailed *P*-value ≤0.05 was considered statistically significant. All the statistical analyses and visualisations were completed with the software RevMan, version 5.3, (Nordic Cochrane Centre, Copenhagen, Denmark), *R*, version 4.1.3 (R Core Team, Vienna, Austria), and *R* packages ‘grid’ (version 4.2.2), ‘forestploter’ (version 0.2.3), ‘pheatmap’ (version 1.0.12), and ‘meta’ (version 6.0.0).

### Study quality score

The quality of the included studies was assessed by the Newcastle-Ottawa Scale (NOS), a validated tool for assessing the quality of non-randomised trials [33]. In the NOS, each study is awarded a maximum score of nine points, and the total score can be divided into three categories – low risk of bias/high quality (0–3 points), medium risk of bias/moderate quality (4–6 points) and high risk of bias/low quality (7–9 points). Team members independently performed the assessment and resolved discrepancies by discussion.

## RESULTS

### Study selection

**Figure 1:**
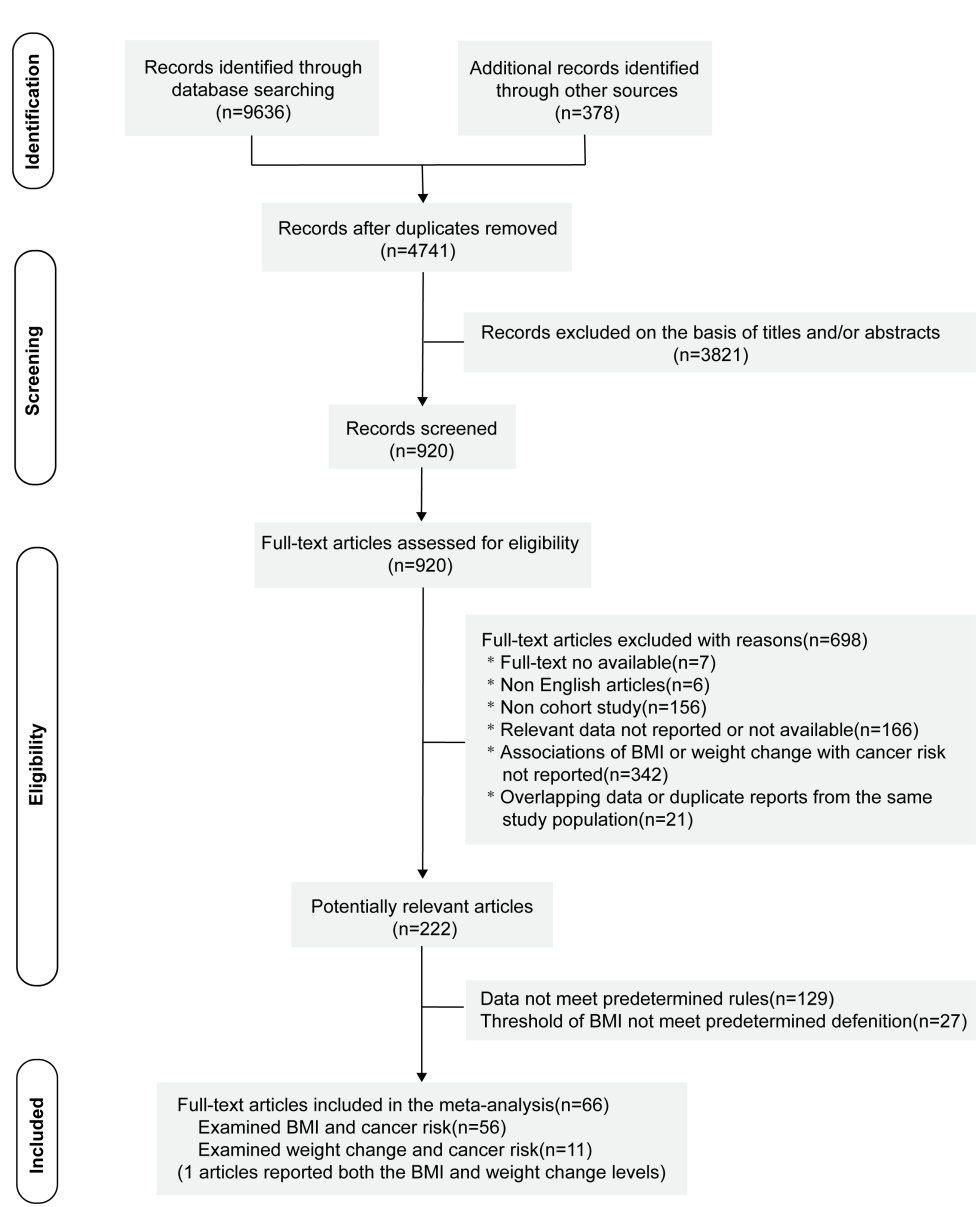
Flow diagram for the search strategy and study selection. BMI – body mass index.

The flowchart of our retrieved and selected studies is presented in **Figure 1**. We obtained 10 014 records during our search, 4741 records remained after removing duplicates and downloading the full text for filtering. After reviewing the titles and abstracts, 3821 records were excluded. The remaining 920 articles were independently assessed for eligibility by three researchers. Of the 920 articles, 698 were excluded for the following reasons: full text was unavailable (n = 7), non-English articles (n = 6), non-cohort studies (n = 156), articles not examining the relationship between BMI or weight change and cancer risk (n = 342), relevant data not reported or unavailable (n = 166), and duplicate data sets (n = 21). After the full-text screening of the remaining 222 articles, 129 were excluded for not meeting the predefined requirements for data, and 27 articles were excluded for not meeting the predefined BMI classification thresholds. Finally, we included 66 studies – 56 for the analysis of BMI and cancer risk and 11 for the analysis of weight change and cancer risk [17,19,34–97].

### Study characteristics

The studies involved approximately 24 million participants and were published between 2004 and 2023. The populations in the studies were predominantly enrolled at ages 40–70 years or older than 18 years, with outcome events including the occurrence of common cancers (such as gastric, colorectal, liver, thyroid, endometrial and breast cancers) and rare cancers (such as diffuse large B-cell lymphoma) (Table S3 in the **Online Supplementary Document**). The proportion of high-quality studies (score ≥7) included in this meta-analysis was approximately 95% (63/66), according to the NOS (Figure S1 in the **Online Supplementary Document**).

### Different BMIs and risk of overall cancer

**Figure 2:**
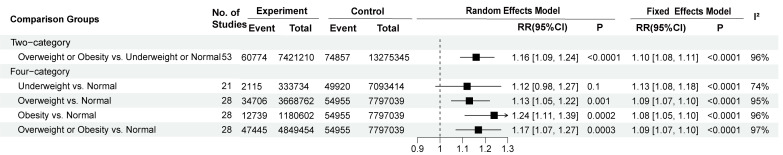
Summary risk estimated by different BMI comparisons for overall cancer incidence. RR – relative risk, CI – confidence interval.

We analysed the risk of overall cancer estimated by different BMI comparisons. Overweight or obesity was associated with an increased risk of overall cancer compared to underweight or normal weight (RR = 1.16; 95% CI = 1.09–1.24, *P* < 0.0001). Furthermore, compared to normal weight, overweight, obesity and overweight or obesity were associated with a greater risk of overall cancer (overweight RR = 1.13; 95% CI = 1.05–1.22, *P* = 0.001; obesity RR = 1.24; 95% CI = 1.11–1.39, *P* = 0.0002; overweight or obesity RR = 1.17; 95% CI = 1.07–1.27, *P* = 0.0003) (**Figure 2**). However, there was no significant correlation between underweight and the risk of overall cancer. When the HRs from three studies were combined, no significant associations were revealed between overweight or obesity and overall cancer risk compared to underweight or normal weight (Figure S2 in the **Online Supplementary Document**).

### Different BMIs and risks of different cancer sites

**Figure 3:**
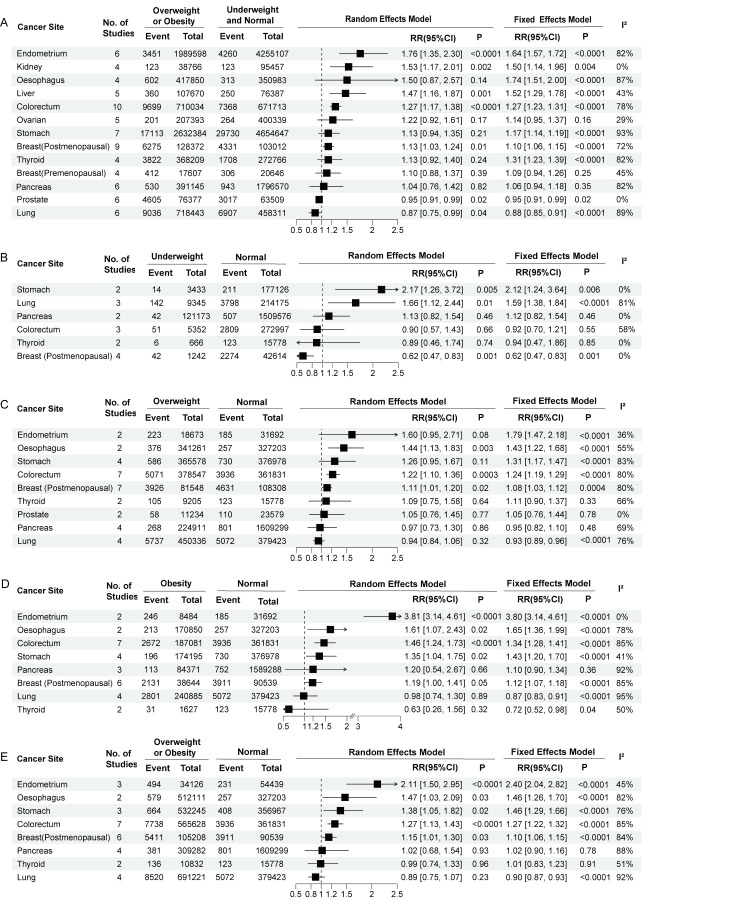
Summary risk estimated by different cancer sites in different BMI comparisons. **Panel A.** Overweight or obesity vs underweight or normal weight. **Panel B.** Underweight vs normal weight. **Panel C.** Overweight vs normal weight. **Panel D.** Obesity vs normal weight. **Panel E.** Overweight or obesity vs normal weight. RR – relative risk, CI – confidence interval.

We analysed the risk of cancer at different sites with different BMIs. Compared to underweight or normal weight, overweight or obesity was associated with an increased risk of endometrial cancer (RR = 1.76; 95% CI = 1.35–2.30, *P* < 0.0001), kidney cancer (RR = 1.50; 95% CI = 1.14–1.96, *P* = 0.004), liver cancer (RR = 1.52; 95% CI = 1.29–1.78, *P* < 0.0001), colorectal cancer (RR = 1.27; 95% CI = 1.17–1.38, *P* < 0.0001) and postmenopausal breast cancer (RR = 1.13; 95% CI = 1.03–1.24, *P* = 0.01). Further, obesity was associated with a reduced risk of prostate cancer (RR = 0.95; 95% CI = 0.91–0.99, *P* = 0.02) and lung cancer (RR = 0.87; 95% CI = 0.75–0.99, *P* = 0.04), and had no significant association with the risk of oesophageal cancer, ovarian cancer, gastric cancer, premenopausal breast cancer, pancreatic cancer or thyroid cancer (**Figure 3**, Panel A).

Compared to a normal weight, underweight was associated with an increased risk of stomach cancer (RR = 2.12; 95% CI = 1.24–3.64, *P* = 0.006) and lung cancer (RR = 1.66; 95% CI = 1.12–2.44, *P* = 0.01), and a reduced risk of postmenopausal breast cancer (RR = 0.62; 95% CI = 0.47–0.83, *P* = 0.001) (**Figure 3**, Panel B). Further, overweight was associated with an increased risk of endometrial cancer (RR = 1.79; 95% CI = 1.47–2.18, *P* < 0.0001), oesophageal cancer (RR = 1.44; 95% CI = 1.13–1.83, *P* = 0.003), colorectal cancer (RR = 1.22; 95% CI = 1.10–1.36, *P* = 0.0003) and postmenopausal breast cancer (RR = 1.11; 95% CI = 1.01–1.20, *P* = 0.02) (**Figure 3**, Panel C).

Compared to normal weight, obesity increased the risk of endometrial cancer (RR = 3.80; 95% CI = 3.14–4.61, *P* < 0.0001), oesophageal cancer (RR = 1.61; 95% CI = 1.07–2.43, *P* = 0.02), colorectal cancer (RR = 1.46; 95% CI = 1.24–1.73, *P* < 0.0001), gastric cancer (RR = 1.43; 95% CI = 1.20–1.70, *P* < 0.0001), and postmenopausal breast cancer (RR = 1.19; 95% CI = 1.00–1.41, *P* = 0.05). Compared to normal weight, overweight or obesity increased the risk of endometrial cancer (RR = 2.40; 95% CI = 2.04–2.82, *P* < 0.0001), oesophageal cancer (RR = 1.47; 95% CI = 1.03–2.09, *P* = 0.03), gastric cancer (RR = 1.38; 95% CI = 1.05–1.82, *P* = 0.02), colorectal cancer (RR = 1.27; 95% CI = 1.13–1.43, *P* < 0.0001), and postmenopausal breast cancer (RR = 1.15; 95% CI = 1.01–1.30, *P* = 0.03) (**Figure 3**, Panels D and E).

When we analysed cancer of the digestive system and female reproductive system separately, we found an increased risk of digestive system cancer in those with overweight (RR = 1.22; 95% CI = 1.13–1.31, *P* < 0.0001), obesity (RR = 1.39; 95% CI = 1.24–1.55, *P* < 0.0001), and overweight or obesity (RR = 1.27; 95% CI = 1.17–1.37, *P* < 0.0001) compared to normal weight. Notably, the risk of cancer in the female reproductive system cancer was reduced in individuals with underweight (RR = 0.70; 95% CI = 0.55–0.88, *P* = 0.002) and increased in individuals with overweight (RR = 1.12; 95% CI = 1.00–1.25, *P* = 0.05), obesity (RR = 1.29; 95% CI = 1.03–1.62, *P* = 0.03), and obesity or overweight (RR = 1.18; 95% CI = 1.02–1.36, *P* = 0.02) (Figure S3 in the **Online Supplementary Document**).

When we further stratified the analysis by sex, we found that underweight, overweight, obesity, and overweight or obesity were associated with an increased risk of colorectal cancer in both men and women, with no significant differences observed between the two groups (Figure S4 in the **Online Supplementary Document**).

Weight change and risks of overall cancer and different cancer sites

**Figure 4:**
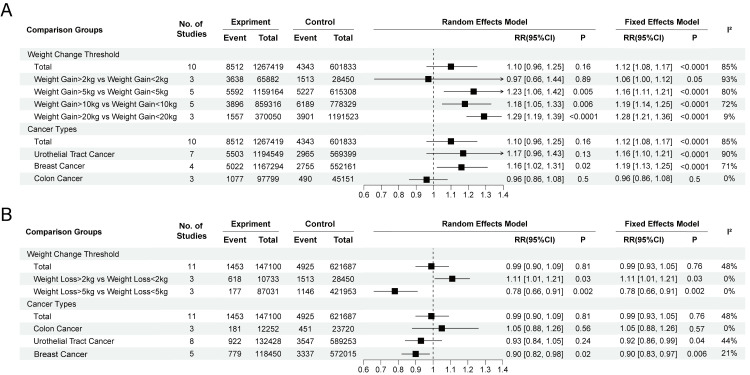
Summary risk estimated by different cancer types and different degrees of weight change. **Panel A.** Weight gain. **Panel B.** Weight loss. RR – relative risk, CI – confidence interval.

In the weight gain vs non-weight change subgroup, individuals who gained >5 kg (RR = 1.23; 95% CI = 1.06–1.42, *P* = 0.005), >10 kg (RR = 1.18; 95% CI = 1.05–1.33, *P* = 0.006), or >20 kg (RR = 1.28; 95% CI = 1.21–1.36, *P* < 0.0001) had a greater risk of developing overall cancer than those who did not experience weight change; while no significant difference in cancer risk was observed between those who gained >2 kg and those who gained any degree of weight (total) (**Figure 4**, Panel A). Regarding cancer type, weight gain was shown to be associated with an increased risk of breast cancer (RR = 1.16; 95% CI = 1.02–1.31, *P* = 0.02) but had no significant effect on the risk of colon or reproductive cancer.

According to the weight loss vs non-weight change analysis, participants who lost >5 kg had a lower risk of overall cancer than those who did not experience weight change (RR = 0.78; 95% CI = 0.66–0.91, *P* = 0.002), while there was no significant change in the risk of overall cancer in those who lost any degree of weight. Those who lost >2 kg had an increased risk of overall cancer (RR = 1.11; 95% CI = 1.01–1.21, *P* = 0.03).

When examining cancer type, weight loss was associated with a decreased risk of breast cancer (RR = 0.90; 95% CI = 0.83–0.97, *P* = 0.006) and reproductive cancer (RR = 0.92; 95% CI = 0.86–0.99, *P* = 0.04) but had no significant effect on colon cancer (RR = 1.05; 95% CI = 0.88–1.26, *P* = 0.57) (**Figure 4**, Panel B).

### Publication bias

In this meta-analysis, we did not find publication bias in the studies on the association between BMI or weight change and the risk of cancer. The funnel plot showed slight symmetry (Figure S5 in the **Online Supplementary Document**), and Egger’s test showed that the following subgroup comparisons all had *P*-values >0.05, indicating no significant publication bias (overweight or obesity vs underweight or normal weight *P* = 0.47; underweight vs normal weight *P* = 0.56; overweight vs normal weight *P* = 0.77; obesity vs normal weight *P* = 0.14; overweight or obesity vs normal weight *P* = 0.47; weight gain vs non-weight change *P* = 0.85) and weight loss vs non-weight change *P* = 0.83).

## DISCUSSION

Our meta-analysis was designed to assess the relationship between BMI or weight change and cancer risk. Our findings revealed that compared to underweight or normal weight, overweight or obesity was significantly associated with an increased risk of endometrial, kidney, liver, colorectal and postmenopausal breast cancer. The association was stronger for an increased risk of endometrial cancer and weaker for liver and colorectal cancers. For prostate cancer and lung cancer patients, compared to underweight or normal weight, overweight or obesity was a protective factor. In addition, a weight gain greater than 5 kg was associated with an increased risk of overall cancer, while a weight loss greater than 5 kg was associated with a decreased risk of overall cancer.

**Figure 5:**
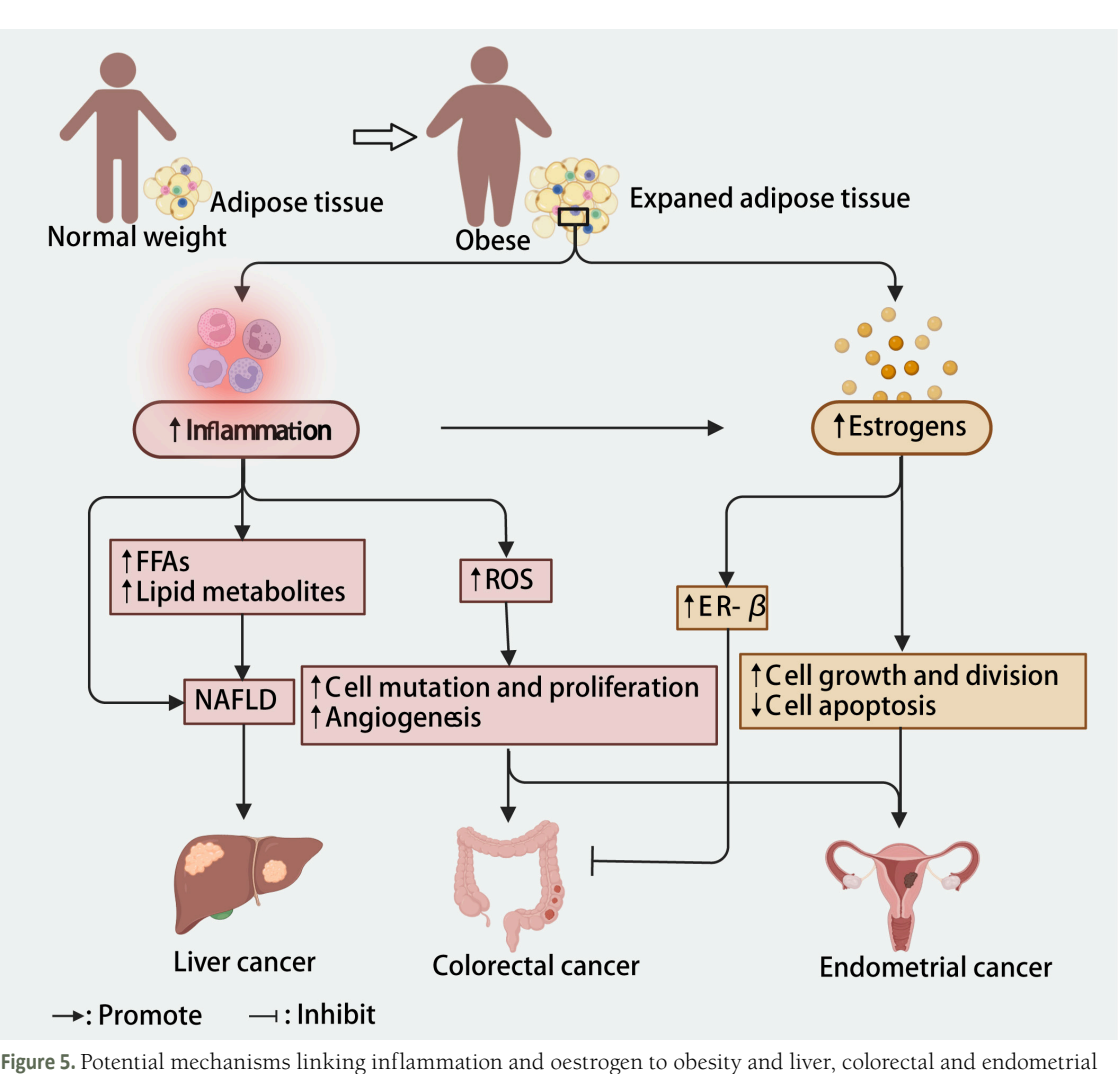
Potential mechanisms linking inflammation and oestrogen to obesity and liver, colorectal and endometrial cancers. Created in Biorender (https://biorender.com/). ER – oestrogen receptor, FFAs – free fatty acids, NAFLD – non-alcoholic fatty liver disease, ROS – reactive oxygen species.

Many studies have described the mechanisms that link overweight or obesity to cancer risk. In the vast majority of tumours, inflammation and oestrogen are the shared mechanisms that increase the risk of cancer due to being overweight and obese. However, the specific role of these mechanisms may vary depending on the tumour type (**Figure 5**). In individuals who are overweight or obese, adipocytes increase in size and release inflammatory factors, leading to chronic low-grade inflammation in the body, which then triggers tumour development and increases cancer risk through a variety of complex mechanisms [98]. In endometrial and colorectal cancers, an increase in inflammatory cytokines can promote the development of tumours by producing damaging reactive oxygen species, causing cell mutation and proliferation, and promoting angiogenesis [98–103]. In the liver, the release of large amounts of inflammatory factors can contribute to the development of non-alcoholic fatty liver disease directly or by increasing the levels of free fatty acids, lipid accumulation, hepatic steatosis and necrosis and causing non-alcoholic fatty liver disease, consequently increasing the risk of liver cancer [98,104]. An obesity-induced proinflammatory environment can cause an imbalance between oestrogen and progesterone, increasing susceptibility to neoplastic processes [105]. Moreover, in adipose tissue, androgens can be converted to oestrogens (estrone and oestradiol) by aromatase enzymes, and the levels of these enzymes increase with increasing BMI [106,107], leading to elevated oestrogen levels in people with overweight or obesity. In endometrial tissue, oestrogen can promote cancer by stimulating cell growth and division and inhibiting apoptosis [108]. Conversely, in the colorectum, oestrogen in women with obesity has been found to reduce the risk of cancer [109]. Increased oestrogen promotes the expression of oestrogen receptor-β, which can promote apoptosis and inhibit carcinogenesis, partially offsetting the increased risk of colorectal cancer caused by obesity [109,110]. Therefore, these differential effects of inflammation and oestrogen in different parts of the body may contribute to the varying associations among the risks of endometrial, liver and colorectal cancer due to being overweight or obese, thus may explain our findings.

The effects of overweight and obesity on cancer risk and their underlying mechanisms differ between tumour types. Our analysis also revealed that, in the case of prostate cancer, being overweight or obese might reduce cancer risk rather than increase it, as observed in other tumour types. This effect may be attributed to the central role of testosterone in the prostate gland. Testosterone is a major androgen in men and is involved in normal prostate growth, and its levels are associated with prostate development and function [111,112]. High testosterone levels can stimulate the proliferation of the prostate tissue [113]. Studies suggest that an increased level of testosterone may be associated with an increased risk of cancer in the prostate [114–116]. In contrast, in individuals with obesity, testosterone levels are typically reduced, which may be detrimental to the growth of tumour cells and tissues in the prostate and thus may lead to a reduced risk of prostate cancer [116–120].

We also found that weight gain over 5 kg was a risk factor for overall cancer, while weight loss over 5 kg was a protective factor. Regarding breast cancer, any degree of weight gain was associated with an increased risk, while weight loss was associated with a decreased risk. The effect of weight change on breast cancer risk may be related to mechanisms such as metabolic dysfunction and oestradiol levels in the body. The metabolic effects of weight gain on breast tissue during adolescence, pregnancy or menopause may increase a woman’s risk of breast cancer [121]. Weight loss has been shown to increase the concentration of sex hormone-binding globulin, a plasma-binding protein with a high specific affinity for oestradiol. Elevated sex hormone-binding globulin levels generally reduce the proportion of oestradiol in the body, thereby reducing the stimulation of breast cell proliferation by oestradiol and thus reducing the risk of cancer [122,123]. In addition, studies have shown that the levels of several cancer biomarkers, including C-reactive protein, tumour necrosis factor-α, interleukin-6, insulin-like growth factor and insulin-like growth factor binding protein, fall rapidly after weight loss [124], suggesting that weight loss can help reduce the risk of cancer.

Our findings point to new directions for weight management in cancer prevention. On the one hand, weight optimisation needs to be tailored for individuals with different BMIs and different cancer risk groups to contribute to cancer risk reduction. For example, in postmenopausal breast cancer patients, underweight was significantly associated with a lower risk, whereas overweight and obesity were associated with a greater risk of occurrence. In contrast, underweight is a risk factor for gastric cancer development. The substantial variability between different BMIs and the risk of cancer development reveals the variability in weight management strategies. On the other hand, since both BMI and weight change were significantly associated with the risk of cancer occurrence, BMI and weight change may contribute to the construction of better cancer risk prediction models.

This study has numerous limitations. First, some results were not significant in the meta-analysis of cancer types, possibly due to the limited size of the data set. Second, the baseline population characteristics were not restricted in this study, and inconsistent baseline population characteristics may also affect the results. Third, due to data limitations, this study did not allow for a comprehensive and systematic analysis of subgroups such as those defined by age and ethnicity. Heterogeneity is often unavoidable in meta-analyses, especially when dealing with diverse baseline population characteristics, such as varying cancer stages among the included patients. Given the difficulty in conducting detailed subgroup analyses due to limited sample sizes and heterogeneous baseline populations, we faced a trade-off between sample size constraints and the potential for extensive subgroup analysis. As such, we endeavoured to investigate scientific outcomes in the presence of certain heterogeneity. Furthermore, to address the issue of heterogeneity and further reduce its impact, obtaining access to raw data for additional analyses would be beneficial. By merging similar patient groups across different studies based on baseline characteristics (e.g. gender, age, ethnicity), we could conduct an in-depth examination of the impact of obesity on cancer risk. However, acquiring such raw data poses considerable challenges and may not be readily achievable. Fourth, this study avoided the inclusion of intentional weight loss-related cohort studies; instead, we opted for studies in which weight change data were obtained via self-reports or third-party institutional measurements. However, there was no way to determine whether the data obtained from self-reports or third-party institutional measures were derived from intentional or unintentional weight loss. Finally, due to the insufficient number of included studies, there was no breakdown of tumour type in the subgroups with a weight gain >5 kg, 10 kg, or 20 kg and weight loss >2 kg or 5 kg, as well as no breakdown of the degree of weight change in the subgroups of breast cancer and reproductive system cancer risk. All of these factors may have contributed to the high heterogeneity of the meta-analysis.

## CONCLUSIONS

In conclusion, this meta-analysis showed that BMI and weight change are associated with cancer risk, with overweight and obesity likely associated with an increased risk of most cancers and weight loss >5 kg likely protective against overall cancer risk. Optimising BMI or weight may be an effective measure for reducing cancer risk. These findings could lead to the development of personalised and more detailed weight management strategies in clinical practice, as well as the optimisation of cancer risk prediction models. More data are needed to investigate the effect of underweight on cancer risk.

## Additional material


Online Supplementary Document.

## References

[1] World Health Organization. Cancer. 2022. Available: https://www.who.int/news-room/fact-sheets/detail/cancer. Accessed: 2 January 2023.

[2] Chhikara BS Parang K Global Cancer Statistics 2022: the trends projection analysis. Chemical Biology Letters. 2023;10:451.

[3] Rawla P Sunkara T Muralidharan P Raj JP Update in global trends and aetiology of hepatocellular carcinoma. Contemp Oncol (Pozn). 2018;22:141–50. 10.5114/wo.2018.78941 30455585 PMC6238087

[4] Hirsch FR Scagliotti GV Mulshine JL Kwon R Curran WJ Wu Y-L Lung cancer: current therapies and new targeted treatments. Lancet. 2017;389:299–311. 10.1016/S0140-6736(16)30958-8 27574741

[5] Bagnardi V Blangiardo M La Vecchia C Corrao G Alcohol consumption and the risk of cancer: a meta-analysis. Alcohol Res Health. 2001;25:263–70. 11910703 PMC6705703

[6] Cowey S Hardy RW The metabolic syndrome: A high-risk state for cancer? Am J Pathol. 2006;169:1505–22. 10.2353/ajpath.2006.051090 17071576 PMC1780220

[7] Hecht SS Cigarette smoking: cancer risks, carcinogens, and mechanisms. Langenbecks Arch Surg. 2006;391:603–13. 10.1007/s00423-006-0111-z 17031696

[8] Shikata K Ninomiya T Kiyohara Y Diabetes mellitus and cancer risk: review of the epidemiological evidence. Cancer Sci. 2013;104:9–14. 10.1111/cas.12043 23066889 PMC7657146

[9] Key TJ Allen NE Spencer EA Travis RC The effect of diet on risk of cancer. Lancet. 2002;360:861–8. 10.1016/S0140-6736(02)09958-0 12243933

[10] Argyrakopoulou G Dalamaga M Spyrou N Kokkinos A Gender Differences in Obesity-Related Cancers. Curr Obes Rep. 2021;10:100–15. 10.1007/s13679-021-00426-0 33523397

[11] Sohn W Lee HW Lee S Lim JH Lee MW Park CH Obesity and the risk of primary liver cancer: A systematic review and meta-analysis. Clin Mol Hepatol. 2021;27:157–74. 10.3350/cmh.2020.0176 33238333 PMC7820201

[12] Vrieling A Buck K Kaaks R Chang-Claude J Adult weight gain in relation to breast cancer risk by estrogen and progesterone receptor status: a meta-analysis. Breast Cancer Res Treat. 2010;123:641–9. 10.1007/s10549-010-1116-4 20711809

[13] Shaw E Farris M McNeil J Friedenreich C Obesity and Endometrial Cancer. Recent Results Cancer Res. 2016;208:107–36. 10.1007/978-3-319-42542-9_7 27909905

[14] Mandic M Safizadeh F Niedermaier T Hoffmeister M Brenner H Association of Overweight, Obesity, and Recent Weight Loss With Colorectal Cancer Risk. JAMA Netw Open. 2023;6:e239556. 10.1001/jamanetworkopen.2023.9556 37083659 PMC10122181

[15] Ellingjord-Dale M Christakoudi S Weiderpass E Panico S Dossus L Olsen A Long-term weight change and risk of breast cancer in the European Prospective Investigation into Cancer and Nutrition (EPIC) study. Int J Epidemiol. 2022;50:1914–26. 10.1093/ije/dyab032 34999853 PMC8743116

[16] Merry AH Schouten LJ Goldbohm RA van den Brandt PA Body mass index, height and risk of adenocarcinoma of the oesophagus and gastric cardia: a prospective cohort study. Gut. 2007;56:1503–11. 10.1136/gut.2006.116665 17337464 PMC2095659

[17] Sanikini H Muller DC Chadeau-Hyam M Murphy N Gunter MJ Cross AJ Anthropometry, body fat composition and reproductive factors and risk of oesophageal and gastric cancer by subtype and subsite in the UK Biobank cohort. PLoS One. 2020;15:e0240413. 10.1371/journal.pone.0240413 33079929 PMC7575071

[18] Lin A Qiu Z Zhang J Luo P Effect of NCOR1 Mutations on Immune Microenvironment and Efficacy of Immune Checkpoint Inhibitors in Patient with Bladder Cancer. Front Immunol. 2021;12:630773. 10.3389/fimmu.2021.630773 33763074 PMC7982737

[19] Kuriyama S Tsubono Y Hozawa A Shimazu T Suzuki Y Koizumi Y Obesity and risk of cancer in Japan. Int J Cancer. 2005;113:148–57. 10.1002/ijc.20529 15386435

[20] Máchová L Cizek L Horakova D Koutna J Lorenc J Janoutova G Association between obesity and cancer incidence in the population of the District Sumperk, Czech Republic. Onkologie. 2007;30:538–42. 17992023 10.1159/000108284

[21] Sjödahl K Jia C Vatten L Nilsen T Hveem K Lagergren J Body mass and physical activity and risk of gastric cancer in a population-based cohort study in Norway. Cancer Epidemiol Biomarkers Prev. 2008;17:135–40. 10.1158/1055-9965.EPI-07-0704 18187390

[22] Yi L Zhang W Zhang H Shen J Zou J Luo P Systematic review and meta-analysis of the benefit of celecoxib in treating advanced non-small-cell lung cancer. Drug Des Devel Ther. 2018;12:2455–66. 10.2147/DDDT.S169627 30122902 PMC6086108

[23] Liu X Sun Q Hou H Zhu K Wang Q Liu H The association between BMI and kidney cancer risk: An updated dose-response meta-analysis in accordance with PRISMA guideline. Medicine (Baltimore). 2018;97:e12860. 10.1097/MD.0000000000012860 30383638 PMC6221676

[24] Moghaddam AA Woodward M Huxley R Obesity and risk of colorectal cancer: a meta-analysis of 31 studies with 70,000 events. Cancer Epidemiol Biomarkers Prev. 2007;16:2533–47. 10.1158/1055-9965.EPI-07-0708 18086756

[25] Qu C Zhang H Cao H Tang L Mo H Liu F Tumor buster - where will the CAR-T cell therapy “missile” go? Mol Cancer. 2022;21:201. 10.1186/s12943-022-01669-8 36261831 PMC9580202

[26] Liu Z Liang Q Ren Y Guo C Ge X Wang L Immunosenescence: molecular mechanisms and diseases. Signal Transduct Target Ther. 2023;8:200. 10.1038/s41392-023-01451-2 37179335 PMC10182360

[27] Hao Y Jiang M Miao Y Li X Hou C Zhang X Effect of long-term weight gain on the risk of breast cancer across women’s whole adulthood as well as hormone-changed menopause stages: A systematic review and dose-response meta-analysis. Obes Res Clin Pract. 2021;15:439–48. 10.1016/j.orcp.2021.08.004 34456166

[28] Hardefeldt PJ Penninkilampi R Edirimanne S Eslick GD Physical Activity and Weight Loss Reduce the Risk of Breast Cancer: A Meta-analysis of 139 Prospective and Retrospective Studies. Clin Breast Cancer. 2018;18:e601–12. 10.1016/j.clbc.2017.10.010 29223719

[29] Zhang X Rhoades J Caan BJ Cohn DE Salani R Noria S Intentional weight loss, weight cycling, and endometrial cancer risk: a systematic review and meta-analysis. Int J Gynecol Cancer. 2019;29:1361–71. 10.1136/ijgc-2019-000728 31451560 PMC6832748

[30] Moher D Liberati A Tetzlaff J Altman DG Grp PRISMA Preferred Reporting Items for Systematic Reviews and Meta-Analyses: The PRISMA Statement. PLoS Med. 2009;6:e1000097. 10.1371/journal.pmed.1000097 19621072 PMC2707599

[31] World Health Organization. Obesity and overweight. 2021. Available: https://www.who.int/news-room/fact-sheets/detail/obesity-and-overweight. Accessed: 2 January 2023.

[32] Jin ZC Zhou XH He J Statistical methods for dealing with publication bias in meta-analysis. Stat Med. 2015;34:343–60. 10.1002/sim.6342 25363575

[33] Wells GA, Shea B, O’Connell D, Peterson J, Welch V, Losos M, et al. The Newcastle-Ottawa Scale (NOS) for assessing the quality of nonrandomised studies in meta-analyses. 2014. Available: https://www.ohri.ca/programs/clinical_epidemiology/oxford.asp. Accessed: 20 March 2024.

[34] Safizadeh F Mandic M Pulte D Niedermaier T Hoffmeister M Brenner H The underestimated impact of excess body weight on colorectal cancer risk: Evidence from the UK Biobank cohort. Br J Cancer. 2023;129:829–37. 10.1038/s41416-023-02351-6 37443347 PMC10449928

[35] Pasqual E O’Brien K Rinaldi S Sandler DP Kitahara CM Obesity, obesity-related metabolic conditions, and risk of thyroid cancer in women: results from a prospective cohort study (Sister Study). Lancet Reg Health Am. 2023;23:100537. 10.1016/j.lana.2023.100537 37346380 PMC10279535

[36] Yang W Zeng X Petrick JL Danford CJ Florio AA Lu B Body mass index trajectories, weight gain and risks of liver and biliary tract cancers. JNCI Cancer Spectr. 2022;6:pkac056. 10.1093/jncics/pkac056 35960613 PMC9406603

[37] Urbute A Frederiksen K Kjaer SK Early adulthood overweight and obesity and risk of premenopausal ovarian cancer, and premenopausal breast cancer including receptor status: prospective cohort study of nearly 500,000 Danish women. Ann Epidemiol. 2022;70:61–7. 10.1016/j.annepidem.2022.03.013 35405346

[38] Song H Jeong A Tran TXM Lee J Kim M Park B Association between Micronutrient Intake and Breast Cancer Risk According to Body Mass Index in South Korean Adult Women: A Cohort Study. Nutrients. 2022;14:2644. 10.3390/nu14132644 35807825 PMC9268499

[39] Shao F Chen Y Xu H Chen X Zhou J Wu Y Metabolic Obesity Phenotypes and Risk of Lung Cancer: A Prospective Cohort Study of 450,482 UK Biobank Participants. Nutrients. 2022;14:3370. 10.3390/nu14163370 36014876 PMC9414360

[40] Park B Associations between obesity, metabolic syndrome, and endometrial cancer risk in East Asian women. J Gynecol Oncol. 2022;33:e35. 10.3802/jgo.2022.33.e35 35320886 PMC9250850

[41] Park B Changes in weight and waist circumference during menopausal transition and postmenopausal breast cancer risk. Int J Cancer. 2022;150:1431–8. 10.1002/ijc.33906 34921731

[42] Nguyen DN Kim JH Kim MK Association of Metabolic Health and Central Obesity with the Risk of Thyroid Cancer: Data from the Korean Genome and Epidemiology Study. Cancer Epidemiol Biomarkers Prev. 2022;31:543–53. 10.1158/1055-9965.EPI-21-0255 34933959

[43] Mao D Lau ESH Wu H Yang A Fan B Shi M Risk Associations of Glycemic Burden and Obesity With Liver Cancer-A 10-Year Analysis of 15,280 Patients With Type 2 Diabetes. Hepatol Commun. 2022;6:1350–60. 10.1002/hep4.1891 35044101 PMC9134801

[44] Lee HW Huang D Shin WK de la Torre K Yang JJ Song M Obesity at early adulthood increases risk of gastric cancer from the Health Examinees-Gem (HEXA-G) study. PLoS One. 2022;17:e0260826. 10.1371/journal.pone.0260826 35120118 PMC8815964

[45] Klintman M Rosendahl AH Randeris B Eriksson M Czene K Hall P Postmenopausal overweight and breast cancer risk; results from the KARMA cohort. Breast Cancer Res Treat. 2022;196:185–96. 10.1007/s10549-022-06664-7 36040641 PMC9550786

[46] Smith SG Sestak I Morris MA Harvie M Howell A Forbes J The impact of body mass index on breast cancer incidence among women at increased risk: an observational study from the International Breast Intervention Studies. Breast Cancer Res Treat. 2021;188:215–23. 10.1007/s10549-021-06141-7 33656637 PMC8233270

[47] Miyata H Shirai K Muraki I Iso H Tamakoshi A Associations of Body Mass Index, Weight Change, Physical Activity, and Sedentary Behavior With Endometrial Cancer Risk Among Japanese Women: The Japan Collaborative Cohort Study. J Epidemiol. 2021;31:621–7. 10.2188/jea.JE20200145 32963209 PMC8593582

[48] Maliniak ML Gapstur SM McCullough LE Rees-Punia E Gaudet MM Um CY Joint associations of physical activity and body mass index with the risk of established excess body fatness-related cancers among postmenopausal women. Cancer Causes Control. 2021;32:127–38. 10.1007/s10552-020-01365-2 33185805

[49] Kim SJ Lubinski J Huzarski T Moller P Armel S Karlan BY Weight Gain and the Risk of Ovarian Cancer in BRCA1 and BRCA2 Mutation Carriers. Cancer Epidemiol Biomarkers Prev. 2021;30:2038–43. 10.1158/1055-9965.EPI-21-0296 34426412

[50] Choi IY Choi YJ Shin DW Han KD Jeon KH Jeong SM Association between obesity and the risk of gastric cancer in premenopausal and postmenopausal women: A nationwide cohort study. J Gastroenterol Hepatol. 2021;36:2834–40. 10.1111/jgh.15558 34033134

[51] Baumeister SE Schlecht I Trabert B Nolde M Meisinger C Leitzmann MF Anthropometric risk factors for ovarian cancer in the NIH-AARP Diet and Health Study. Cancer Causes Control. 2021;32:231–9. 10.1007/s10552-020-01377-y 33481137 PMC7870624

[52] Abe SK Narita S Saito E Sawada N Shimazu T Goto A Body Mass Index, Height, Weight Change, and Subsequent Lung Cancer Risk: The Japan Public Health Center-Based Prospective Study. Cancer Epidemiol Biomarkers Prev. 2021;30:1708–16. 10.1158/1055-9965.EPI-21-0195 34172462

[53] Sanikini H Muller DC Sophiea M Rinaldi S Agudo A Duell EJ Anthropometric and reproductive factors and risk of esophageal and gastric cancer by subtype and subsite: Results from the European Prospective Investigation into Cancer and Nutrition (EPIC) cohort. Int J Cancer. 2020;146:929–42. 10.1002/ijc.32386 31050823 PMC6973006

[54] Renehan AG Pegington M Harvie MN Sperrin M Astley SM Brentnall AR Young adulthood body mass index, adult weight gain and breast cancer risk: the PROCAS Study (United Kingdom). Br J Cancer. 2020;122:1552–61. 10.1038/s41416-020-0807-9 32203222 PMC7217761

[55] Minami CA Zabor EC Gilbert E Newman A Park A Jochelson MS Do Body Mass Index and Breast Density Impact Cancer Risk Among Women with Lobular Carcinoma In Situ? Ann Surg Oncol. 2020;27:1844–51. 10.1245/s10434-019-08126-9 31898097 PMC7211554

[56] Luo J Chen X Manson JE Shadyab AH Wactawski-Wende J Vitolins M Birth weight, weight over the adult life course and risk of breast cancer. Int J Cancer. 2020;147:65–75. 10.1002/ijc.32710 31584193

[57] Zohar L Rottenberg Y Twig G Katz L Leiba A Derazne E Adolescent overweight and obesity and the risk for pancreatic cancer among men and women: a nationwide study of 1.79 million Israeli adolescents. Cancer. 2019;125:118–26. 10.1002/cncr.31764 30417331

[58] Wakamatsu M Sugawara Y Zhang S Tanji F Tomata Y Tsuji I Weight change since age 20 and incident risk of obesity-related cancer in Japan: a pooled analysis of the Miyagi Cohort Study and the Ohsaki Cohort Study. Int J Cancer. 2019;144:967–80. 10.1002/ijc.31743 29992563 PMC6587529

[59] Kawachi A Shimazu T Budhathoki S Sawada N Yamaji T Iwasaki M Association of BMI and height with the risk of endometrial cancer, overall and by histological subtype: a population-based prospective cohort study in Japan. Eur J Cancer Prev. 2019;28:196–202. 10.1097/CEJ.0000000000000449 29672353

[60] Hirabayashi M Inoue M Sawada N Saito E Abe SK Hidaka A Effect of body-mass index on the risk of gastric cancer: A population-based cohort study in A Japanese population. Cancer Epidemiol. 2019;63:101622. 10.1016/j.canep.2019.101622 31654882

[61] Everatt R Virviciute D Tamosiunas A Body mass index and other risk factors for kidney cancer in men: a cohort study in Lithuania. Cent Eur J Public Health. 2019;27:272–8. 10.21101/cejph.a5080 31951685

[62] Xu HL Zhang ML Yan YJ Fang F Guo Q Xu DL Body mass index and cancer risk among Chinese patients with type 2 diabetes mellitus. BMC Cancer. 2018;18:795. 10.1186/s12885-018-4675-0 30081866 PMC6080536

[63] Schoemaker MJ Nichols HB Wright LB Brook MN Jones ME O’Brien KM Association of Body Mass Index and Age With Subsequent Breast Cancer Risk in Premenopausal Women. JAMA Oncol. 2018;4:e181771. 10.1001/jamaoncol.2018.1771 29931120 PMC6248078

[64] Kim SJ Huzarski T Gronwald J Singer CF Moller P Lynch HT Prospective evaluation of body size and breast cancer risk among BRCA1 and BRCA2 mutation carriers. Int J Epidemiol. 2018;47:987–97. 10.1093/ije/dyy039 29547931 PMC6005062

[65] da Silva M Weiderpass E Licaj I Lissner L Rylander C Excess body weight, weight gain and obesity-related cancer risk in women in Norway: the Norwegian Women and Cancer study. Br J Cancer. 2018;119:646–56. 10.1038/s41416-018-0240-5 30202086 PMC6162329

[66] Chadid S Singer MR Kreger BE Bradlee ML Moore LL Midlife weight gain is a risk factor for obesity-related cancer. Br J Cancer. 2018;118:1665–71. 10.1038/s41416-018-0106-x 29895939 PMC6008441

[67] Shin CM Han K Lee DH Choi YJ Kim N Park YS Association Among Obesity, Metabolic Health, and the Risk for Colorectal Cancer in the General Population in Korea Using the National Health Insurance Service-National Sample Cohort. Dis Colon Rectum. 2017;60:1192–200. 10.1097/DCR.0000000000000876 28991084

[68] Rosner B Eliassen AH Toriola AT Chen WY Hankinson SE Willett WC Weight and weight changes in early adulthood and later breast cancer risk. Int J Cancer. 2017;140:2003–14. 10.1002/ijc.30627 28133728 PMC5798241

[69] Leiba A Duek A Afek A Derazne E Leiba M Obesity and related risk of myeloproliferative neoplasms among israeli adolescents. Obesity (Silver Spring). 2017;25:1187–90. 10.1002/oby.21863 28500663

[70] Dickerman BA Ahearn TU Giovannucci E Stampfer MJ Nguyen PL Mucci LA Weight change, obesity and risk of prostate cancer progression among men with clinically localized prostate cancer. Int J Cancer. 2017;141:933–44. 10.1002/ijc.30803 28543830 PMC5518616

[71] Yamamoto-Honda R Takahashi Y Yoshida Y Kwazu S Iwamoto Y Kajio H Body mass index and the risk of cancer incidence in patients with type 2 diabetes in Japan: Results from the National Center Diabetes Database. J Diabetes Investig. 2016;7:908–14. 10.1111/jdi.12522 27181076 PMC5089955

[72] Sponholtz TR Palmer JR Rosenberg L Hatch EE Adams-Campbell LL Wise LA Body Size, Metabolic Factors, and Risk of Endometrial Cancer in Black Women. Am J Epidemiol. 2016;183:259–68. 10.1093/aje/kwv186 26823438 PMC4753280

[73] White AJ Nichols HB Bradshaw PT Sandler DP Overall and central adiposity and breast cancer risk in the Sister Study. Cancer. 2015;121:3700–8. 10.1002/cncr.29552 26193782 PMC4592412

[74] Møller H Roswall N Van Hemelrijck M Larsen SB Cuzick J Holmberg L Prostate cancer incidence, clinical stage and survival in relation to obesity: a prospective cohort study in Denmark. Int J Cancer. 2015;136:1940–7. 10.1002/ijc.29238 25264293

[75] de Mutsert R Sun Q Willett WC Hu FB van Dam RM Overweight in early adulthood, adult weight change, and risk of type 2 diabetes, cardiovascular diseases, and certain cancers in men: a cohort study. Am J Epidemiol. 2014;179:1353–65. 10.1093/aje/kwu052 24786797 PMC4036209

[76] Patel AV Diver WR Teras LR Birmann BM Gapstur SM Body mass index, height and risk of lymphoid neoplasms in a large United States cohort. Leuk Lymphoma. 2013;54:1221–7. 10.3109/10428194.2012.742523 23098244

[77] Lam TK Moore SC Brinton LA Smith L Hollenbeck AR Gierach GL Anthropometric measures and physical activity and the risk of lung cancer in never-smokers: a prospective cohort study. PLoS One. 2013;8:e70672. 10.1371/journal.pone.0070672 23940620 PMC3734257

[78] Alsaker MD Janszky I Opdahl S Vatten LJ Romundstad PR Weight change in adulthood and risk of postmenopausal breast cancer: the HUNT study of Norway. Br J Cancer. 2013;109:1310–7. 10.1038/bjc.2013.403 23880822 PMC3778278

[79] Smith L Brinton LA Spitz MR Lam TK Park Y Hollenbeck AR Body mass index and risk of lung cancer among never, former, and current smokers. J Natl Cancer Inst. 2012;104:778–89. 10.1093/jnci/djs179 22457475 PMC3352831

[80] Renehan AG Flood A Adams KF Olden M Hollenbeck AR Cross AJ Body mass index at different adult ages, weight change, and colorectal cancer risk in the National Institutes of Health-AARP Cohort. Am J Epidemiol. 2012;176:1130–40. 10.1093/aje/kws192 23186750 PMC6287246

[81] Cecchini RS Costantino JP Cauley JA Cronin WM Wickerham DL Land SR Body mass index and the risk for developing invasive breast cancer among high-risk women in NSABP P-1 and STAR breast cancer prevention trials. Cancer Prev Res (Phila). 2012;5:583–92. 10.1158/1940-6207.CAPR-11-0482 22318751 PMC4131545

[82] Chamberlain C Romundstad P Vatten L Gunnell D Martin RM The association of weight gain during adulthood with prostate cancer incidence and survival: a population-based cohort. Int J Cancer. 2011;129:1199–206. 10.1002/ijc.25739 21064096

[83] Sawada N Inoue M Sasazuki S Iwasaki M Yamaji T Shimazu T Body mass index and subsequent risk of kidney cancer: a prospective cohort study in Japan. Ann Epidemiol. 2010;20:466–72. 10.1016/j.annepidem.2010.03.008 20470974

[84] Laake I Thune I Selmer R Tretli S Slattery ML Veierod MB A prospective study of body mass index, weight change, and risk of cancer in the proximal and distal colon. Cancer Epidemiol Biomarkers Prev. 2010;19:1511–22. 10.1158/1055-9965.EPI-09-0813 20501754

[85] Bassett JK Severi G English DR Baglietto L Krishnan K Hopper JL Body size, weight change, and risk of colon cancer. Cancer Epidemiol Biomarkers Prev. 2010;19:2978–86. 10.1158/1055-9965.EPI-10-0543 20870733

[86] Rod NH Hansen AM Nielsen J Schnohr P Gronbaek M Low-risk factor profile, estrogen levels, and breast cancer risk among postmenopausal women. Int J Cancer. 2009;124:1935–40. 10.1002/ijc.24136 19123466

[87] Mijović T How J Pakdaman M Rochon L Gologan O Hier MP Body mass index in the evaluation of thyroid cancer risk. Thyroid. 2009;19:467–72. 10.1089/thy.2008.0386 19415996

[88] Leitzmann MF Koebnick C Danforth KN Brinton LA Moore SC Hollenbeck AR Body mass index and risk of ovarian cancer. Cancer. 2009;115:812–22. 10.1002/cncr.24086 19127552 PMC3507338

[89] Thygesen LC Gronbaek M Johansen C Fuchs CS Willett WC Giovannucci E Prospective weight change and colon cancer risk in male US health professionals. Int J Cancer. 2008;123:1160–5. 10.1002/ijc.23612 18546286 PMC3965300

[90] Rodriguez C Freedland SJ Deka A Jacobs EJ McCullough ML Patel AV Body mass index, weight change, and risk of prostate cancer in the Cancer Prevention Study II Nutrition Cohort. Cancer Epidemiol Biomarkers Prev. 2007;16:63–9. 10.1158/1055-9965.EPI-06-0754 17179486

[91] Nöthlings U Wilkens LR Murphy SP Hankin JH Henderson BE Kolonel LN Body mass index and physical activity as risk factors for pancreatic cancer: the Multiethnic Cohort Study. Cancer Causes Control. 2007;18:165–75. 10.1007/s10552-006-0100-0 17219012

[92] Iwasaki M Otani T Inoue M Sasazuki S Tsugane S Japan Public Health Center-Based Prospective Study G. Body size and risk for breast cancer in relation to estrogen and progesterone receptor status in Japan. Ann Epidemiol. 2007;17:304–12. 10.1016/j.annepidem.2006.09.003 17174568

[93] N’Kontchou G Paries J Htar MT Ganne-Carrie N Costentin L Grando-Lemaire V Risk factors for hepatocellular carcinoma in patients with alcoholic or viral C cirrhosis. Clin Gastroenterol Hepatol. 2006;4:1062–8. 10.1016/j.cgh.2006.05.013 16844421

[94] Patel AV Rodriguez C Bernstein L Chao A Thun MJ Calle EE Obesity, recreational physical activity, and risk of pancreatic cancer in a large U.S. Cohort. Cancer Epidemiol Biomarkers Prev. 2005;14:459–66. 10.1158/1055-9965.EPI-04-0583 15734973

[95] Niwa Y Yatsuya H Tamakoshi K Nishio K Kondo T Lin Y Relationship between body mass index and the risk of ovarian cancer in the Japanese population: findings from the Japanese Collaborate Cohort (JACC) study. J Obstet Gynaecol Res. 2005;31:452–8. 10.1111/j.1447-0756.2005.00319.x 16176517

[96] Moore LL Bradlee ML Singer MR Splansky GL Proctor MH Ellison RC BMI and waist circumference as predictors of lifetime colon cancer risk in Framingham Study adults. Int J Obes Relat Metab Disord. 2004;28:559–67. 10.1038/sj.ijo.0802606 14770200

[97] Lin J Zhang SM Cook NR Rexrode KM Lee IM Buring JE Body mass index and risk of colorectal cancer in women (United States). Cancer Causes Control. 2004;15:581–9. 10.1023/B:CACO.0000036168.23351.f1 15280637

[98] Kolb R Sutterwala FS Zhang W Obesity and cancer: inflammation bridges the two. Curr Opin Pharmacol. 2016;29:77–89. 10.1016/j.coph.2016.07.005 27429211 PMC4992602

[99] Pérez-Martín AR Castro-Eguiluz D Cetina-Perez L Velasco-Torres Y Bahena-Gonzalez A Montes-Servin E Impact of metabolic syndrome on the risk of endometrial cancer and the role of lifestyle in prevention. Bosn J Basic Med Sci. 2022;22:499–510. 10.17305/bjbms.2021.6963 35276057 PMC9392984

[100] Bou Malhab LJ Abdel-Rahman WM Obesity and Inflammation: Colorectal Cancer Engines. Curr Mol Pharmacol. 2022;15:620–46. 10.2174/1874467214666210906122054 34488607

[101] Agnew HJ Kitson SJ Crosbie EJ Gynecological malignancies and obesity. Best Pract Res Clin Obstet Gynaecol. 2023;88:102337. 10.1016/j.bpobgyn.2023.102337 37117071

[102] Chen N Lu B Fu Y Autophagic Clearance of Lipid Droplets Alters Metabolic Phenotypes in a Genetic Obesity-Diabetes Mouse Model. Phenomics. 2022;3:119–29. 10.1007/s43657-022-00080-z 37197643 PMC10110819

[103] He Y Li J Yue T Zheng W Guo Y Zhang H Seasonality and Sex-Biased Fluctuation of Birth Weight in Tibetan Populations. Phenomics. 2022;2:64–71. 10.1007/s43657-021-00038-7 36939792 PMC9590487

[104] Alzahrani B Iseli TJ Hebbard LW Non-viral causes of liver cancer: does obesity led inflammation play a role? Cancer Lett. 2014;345:223–9. 10.1016/j.canlet.2013.08.036 24007864

[105] Modugno F Ness RB Chen C Weiss NS Inflammation and endometrial cancer: a hypothesis. Cancer Epidemiol Biomarkers Prev. 2005;14:2840–7. 10.1158/1055-9965.EPI-05-0493 16364998

[106] Key TJ Appleby PN Reeves GK Roddam A Dorgan JF Longcope C Body mass index, serum sex hormones, and breast cancer risk in postmenopausal women. J Natl Cancer Inst. 2003;95:1218–26. 10.1093/jnci/djg022 12928347

[107] Schmandt RE Iglesias DA Co NN Lu KH Understanding obesity and endometrial cancer risk: opportunities for prevention. Am J Obstet Gynecol. 2011;205:518–25. 10.1016/j.ajog.2011.05.042 21802066 PMC4264838

[108] Kaaks R Lukanova A Kurzer MS Obesity, endogenous hormones, and endometrial cancer risk: a synthetic review. Cancer Epidemiol Biomarkers Prev. 2002;11:1531–43. 12496040

[109] Kim H Giovannucci EL Sex differences in the association of obesity and colorectal cancer risk. Cancer Causes Control. 2017;28:1–4. 10.1007/s10552-016-0831-5 27878394

[110] Chen J Iverson D Estrogen in obesity-associated colon cancer: friend or foe? Protecting postmenopausal women but promoting late-stage colon cancer. Cancer Causes Control. 2012;23:1767–73. 10.1007/s10552-012-0066-z 23011535

[111] Nassar GN, Leslie SW. Physiology, testosterone. Treasure Island (FL): StatPearls Publishing; 2024. 30252384

[112] Isaacs JT. Testosterone and the prostate. In: Nieschlag E, Behre HM, editors. Testosterone: Action, Deficiency, Substitution. Cambridge, UK: Cambridge University Press; 2004.

[113] Pollard M Luckert PH Schmidt MA Induction of prostate adenocarcinomas in Lobund Wistar rats by testosterone. Prostate. 1982;3:563–8. 10.1002/pros.2990030605 7155989

[114] Loh NY Wang W Noordam R Christodoulides C Obesity, Fat Distribution and Risk of Cancer in Women and Men: A Mendelian Randomisation Study. Nutrients. 2022;14:5259. 10.3390/nu14245259 36558416 PMC9784937

[115] Parsons JK Carter HB Platz EA Wright EJ Landis P Metter EJ Serum testosterone and the risk of prostate cancer: potential implications for testosterone therapy. Cancer Epidemiol Biomarkers Prev. 2005;14:2257–60. 10.1158/1055-9965.EPI-04-0715 16172240

[116] Watts EL Appleby PN Perez-Cornago A Bueno-de-Mesquita HB Chan JM Chen C Low Free Testosterone and Prostate Cancer Risk: A Collaborative Analysis of 20 Prospective Studies. Eur Urol. 2018;74:585–94. 10.1016/j.eururo.2018.07.024 30077399 PMC6195673

[117] Kanabar R Mazur A Plum A Schmied J Correlates of testosterone change as men age. Aging Male. 2022;25:29–40. 10.1080/13685538.2021.2023493 34983291

[118] Wright ME Chang SC Schatzkin A Albanes D Kipnis V Mouw T Prospective study of adiposity and weight change in relation to prostate cancer incidence and mortality. Cancer. 2007;109:675–84. 10.1002/cncr.22443 17211863

[119] Wu H Liu Y Wang J Chen S Xie L Wu X Schizophrenia and obesity: May the gut microbiota serve as a link for the pathogenesis? iMeta. 2023;2:e99. 10.1002/imt2.99 PMC10989809 38868440

[120] Liu L Xie Y Yang H Lin A Dong M Wang H HPVTIMER: A shiny web application for tumor immune estimation in human papillomavirus-associated cancers. iMeta. 2023;2:e130. 10.1002/imt2.130 PMC10989930 38867938

[121] Stoll BA Timing of weight gain in relation to breast cancer risk. Ann Oncol. 1995;6:245–8. 10.1093/oxfordjournals.annonc.a059153 7612489

[122] Calle EE Kaaks R Overweight, obesity and cancer: epidemiological evidence and proposed mechanisms. Nat Rev Cancer. 2004;4:579–91. 10.1038/nrc1408 15286738

[123] Zeleniuch-Jacquotte A Shore RE Koenig KL Akhmedkhanov A Afanasyeva Y Kato I Postmenopausal levels of oestrogen, androgen, and SHBG and breast cancer: long-term results of a prospective study. Br J Cancer. 2004;90:153–9. 10.1038/sj.bjc.6601517 14710223 PMC2395327

[124] Byers T Sedjo RL Does intentional weight loss reduce cancer risk? Diabetes Obes Metab. 2011;13:1063–72. 10.1111/j.1463-1326.2011.01464.x 21733057

