# Supplementary material for: Role of body mass index and weight change in the risk of cancer: A systematic review and meta-analysis of 66 cohort studies
Source: J Glob Health. 2024 Mar 29;14:04067. doi: 10.7189/jogh.14.04067 (PMC10978059; doi:10.7189/jogh.14.04067)

ONLINE SUPPLEMENTARY DOCUMENT

Title: Role of body mass index and weight change in the risk of cancer: A systematic review and meta-analysis of 66 cohort studies.

Table S1. PRISMA 2009 Checklist

PRISMA 2009 Checklist

Section/topic	#	Checklist item	Reported on page #
**TITLE**			
Title	1	Identify the report as a systematic review, meta-analysis, or both.	1
**ABSTRACT**			
Structured summary	2	Provide a structured summary including, as applicable: background; objectives; data sources; study eligibility criteria, participants, and interventions; study appraisal and synthesis methods; results; limitations; conclusions and implications of key findings; systematic review registration number.	3-4
**INTRODUCTION**			
Rationale	3	Describe the rationale for the review in the context of what is already known.	4-6
Objectives	4	Provide an explicit statement of questions being addressed with reference to participants, interventions, comparisons, outcomes, and study design (PICOS).	5-6
**METHODS**			
Protocol and registration	5	Indicate if a review protocol exists, if and where it can be accessed (e.g., Web address), and, if available, provide registration information including registration number.	6
Eligibility criteria	6	Specify study characteristics (e.g., PICOS, length of follow-up) and report characteristics (e.g., years considered, language, publication status) used as criteria for eligibility, giving rationale.	7
Information sources	7	Describe all information sources (e.g., databases with dates of coverage, contact with study authors to identify additional studies) in the search and date last searched.	6
Search	8	Present full electronic search strategy for at least one database, including any limits used, such that it could be repeated.	6
Study selection	9	State the process for selecting studies (i.e., screening, eligibility, included in systematic review, and, if applicable, included in the meta-analysis).	7
Data collection process	10	Describe method of data extraction from reports (e.g., piloted forms, independently, in duplicate) and any processes for obtaining and confirming data from investigators.	7
Data items	11	List and define all variables for which data were sought (e.g., PICOS, funding sources) and any assumptions and simplifications made.	7
Risk of bias in individual studies	12	Describe methods used for assessing risk of bias of individual studies (including specification of whether this was done at the study or outcome level), and how this information is to be used in any data synthesis.	8-9
Summary measures	13	State the principal summary measures (e.g., risk ratio, difference in means).	7
Synthesis of results	14	Describe the methods of handling data and combining results of studies, if done, including measures of consistency (e.g., I^2) for each meta-analysis.	7-8

Section/topic	#	Checklist item	Reported on page #
Risk of bias across studies	15	Specify any assessment of risk of bias that may affect the cumulative evidence (e.g., publication bias, selective reporting within studies).	8
Additional analyses	16	Describe methods of additional analyses (e.g., sensitivity or subgroup analyses, meta-regression), if done, indicating which were pre-specified.	7-8
**RESULTS**			
Study selection	17	Give numbers of studies screened, assessed for eligibility, and included in the review, with reasons for exclusions at each stage, ideally with a flow diagram.	9-10
Study characteristics	18	For each study, present characteristics for which data were extracted (e.g., study size, PICOS, follow-up period) and provide the citations.	10-11
Risk of bias within studies	19	Present data on risk of bias of each study and, if available, any outcome level assessment (see item 12).	11
Results of individual studies	20	For all outcomes considered (benefits or harms), present, for each study: (a) simple summary data for each intervention group (b) effect estimates and confidence intervals, ideally with a forest plot.	8
Synthesis of results	21	Present results of each meta-analysis done, including confidence intervals and measures of consistency.	9-17
Risk of bias across studies	22	Present results of any assessment of risk of bias across studies (see Item 15).	17
Additional analysis	23	Give results of additional analyses, if done (e.g., sensitivity or subgroup analyses, meta-regression [see Item 16]).	12-16
**DISCUSSION**			
Summary of evidence	24	Summarize the main findings including the strength of evidence for each main outcome; consider their relevance to key groups (e.g., healthcare providers, users, and policy makers).	17-21
Limitations	25	Discuss limitations at study and outcome level (e.g., risk of bias), and at review-level (e.g., incomplete retrieval of identified research, reporting bias).	21-22
Conclusions	26	Provide a general interpretation of the results in the context of other evidence, and implications for future research.	22-23
**FUNDING**			
Funding	27	Describe sources of funding for the systematic review and other support (e.g., supply of data); role of funders for the systematic review.	23

From: Moher D, Liberati A, Tetzlaff J, Altman DG, The PRISMA Group (2009). Preferred Reporting Items for Systematic Reviews and Meta-Analyses: The PRISMA Statement. PLoS Med 6(7): e1000097. doi:10.1371/journal.pmed1000097

For more information, visit: **www.prisma-statement.org**.

Table S2. Search Strategy

Database	Search strategy
Pubmed	(cancer[Title/Abstract] or carcinoma[Title/Abstract]) AND (risk[Title]) AND (weight[Title/Abstract] or overweight[Title/Abstract] or obesity[Title/Abstract])
Web of Science	(TI=(obesity) OR TI=(overweight) OR TI=(weight)) AND (TI=(carcinoma) OR TI=(cancer)) AND (TI=(risk))

Table S3. Characteristics of the studies included in the meta-analysis.

Study	Year	Country or Region	No. of Participants	Sex（M/F）	Age at entry（years）	Outcome	Study Quality	Included Data Type of Cancer incidence	Figure			Supplementary Figure		
									2	3	4	2	3	4
Pasqual E, 2023[1]	2023	USA	47739	F	35-74	Thyroid cancer	8	Event/Total	√	√				
Safizadeh F, 2023[2]	2023	UK	453049	M/F	40-69	Colorectal cancer	7	Event/Total	√	√				
Nguyen DN, 2022[3]	2022	Korea	160650	M/F	40-79	Thyroid Cancer	8	Event/Total	√	√				
Lee HW,2022[4]	2022	Korea	134130	M/F	40-69	Gastric Cancer	9	Event/Total	√					
Mao D, 2022[5]	2022	Hong Kong SAR, China	15281	M/F	NA	Liver Cancer	6	Event/Total	√	√				
Miyata H, 2021[6]	2021	Japan	33,801	M/F	40-79	Endometrial Cancer	9	Event/Total	√	√	√		√	
Abe SK,2021[7]	2021	Japan	92098	M/F	40-69	Liver Cancer	8	Event/Total	√	√				
Smith SG, 2021[8]	2021	United Kingdom	10653	M/F	35–70	Breast Cancer	8	Event/Total	√	√				
Baumeister SE, 2021[9]	2021	Germany	145575	F	50-72	Ovarian Cancer	8	HR				√		
Maliniak ML, 2021[10]	2021	USA	43795	F	63.5 (mean)	Breast Cancer, Endometrial Cancer, Colon Cancer	9	Event/Total	√	√			√	
Sanikini H, 2020[11]	2020	United Kingdom	458713	M/F	40-69	Esophageal Adenocarcinoma	9	Event/Total	√	√			√	
Luo J,2020[12]	2020	USA	70379	F	50-79	Breast Cancer	9	Event/Total	√	√			√	
Minami CA, 2020[13]	2020	USA	1222	M/F	NA	Lobular Carcinoma In Situ	4	HR				√		
Renehan AG, 2020[14]	2020	United Kingdom	47042	F	47-73	Breast Cancer	8	Event/Total	√	√				
Sanikini H, 2020[15]	2020	United Kingdom	476160	M/F	25-70	Esophagus Cancer, Gastric Cancer	9	Event/Total	√	√			√	

Everatt R, 2019[16]	2019	Lithuania	6849	M	45-59	Kidney Cancer	8	Event/Total	√	√		
Hirabayashi M, 2019[17]	2019	Japan	92056	M/F	40-69	Gastric Cancer	9	Event/Total	√	√		
Kawachi A, 2019[18]	2019	Japan	53651	F	40-69	Endometrial Cancer	9	Event/Total	√	√		
Wakamatsu M, 2019[19]	2019	Japan	78743	M/F	40-79	Obesity Related Cancer	9	Event/Total	√			
Zohar L, 2019[20]	2019	Israel	1794570	M/F	16-19	Pancreatic Cancer	8	Event/Total	√	√		√
Schoemaker MJ, 2018[21]	2018	United Kingdom	758592	F	40-69	Breast Cancer	8	Event/Total	√			
Xu HL, 2018[22]	2018	People's Republic of China	51004	M/F	61.39(mean)	Stomach, Colorectal, Pancreas, Trachea, Bronchus & Lung, Breast, Prostate , Bladder, Thyroid Cancer	9	Event/Total	√	√		√
Dickerman BA, 2017[23]	2017	USA	5158	M	40-75	Prostatic Cancer	9	Event/Total	√	√		
Leiba A, 2017[24]	2017	Israel	2516256	M/F	16-19	Bone Marrow Proliferative Tumor （MPN）	7	HR			√	
Yamamoto-Honda R, 2016[25]	2016	Japan	2334	M/F	62.0 （median）	Oropharynx, Submandibular Gland, Larynx, Esophageal (Squamous Cell Cancer), Stomach, Duodenum, Colorectal, Liver, Gall Bladder, Biliary Tract, Pancreas, Lung, Breast (Postmenopausal), Endometrial, Ovary,	9	Event/Total	√	√		

						Prostate, Kidney, Renal Pelvis, Urinary Bladder, Hematopoietic, Gastric Gastrointestinal Stromal Tumor								
Sponholtz TR, 2016[26]	2016	USA	47557	F	21-69	Endometrial Cancer	9	Event/Total	√					
White AJ,2015[27]	2015	USA	50884	F	35-74	Breast Cancer	9	Event/Total	√				√	
Moller H, 2015[28]	2015	United Kingdom	26944	M	50-64	Prostatic Cancer	8	Event/Total	√	√				
de Mutsert R, 2014[29]	2014	Singapore	39909	M/F	40-75	Obesity-related Cancer	8	Event/Total	√					
Lam TK, 2013[30]	2013	USA	158415	M/F	50-71	Lung Cancer	8	Event/Total	√	√				
Patel AV, 2013[31]	2013	USA	152423	M/F	50-74	Diffuse Large B cell Lymphoma	8	Event/Total	√					
Renehan AG, 2012[32]	2012	United Kingdom	273679	M/F	50-71	Colorectal Cancer	8	Event/Total	√	√			√	√
Smith L, 2012[33]	2012	USA	448732	M/F	50-71	Lung Cancer	9	Event/Total	√	√				
Cecchini RS, 2012[34]	2012	USA	12243	F	NA	Breast Cancer	8	Event/Total	√	√				
Laake I, 2010[35]	2010	Norway	21687	M/F	35-49	Colon Cancer	9	Event/Total	√	√			√	√
Sawada N, 2010[36]	2010	Japan	99462	M/F	40-59	Kidney Cancer	9	Event/Total	√	√				
Mijovic T, 2009[37]	2009	Canada	253	M/F	19-85	Cancer of Thyroid gland	4	Event/Total	√	√				
Rod NH, 2009[38]	2009	Denmark	5054	F	62（mean）	Breast Cancer	9	Event/Total	√	√			√	
Leitzmann MF, 2009[39]	2009	USA	94525	F	50-71	Ovarian Cancer	7	Event/Total	√	√				

Iwasaki M, 2007[40]	2007	Japan	55537	F	40-69	Breast Cancer	9	Event/Total	√				
Nothlings U, 2007[41]	2007	USA	167430	M/F	45-75	Prostatic Cancer	9	Event/Total	√	√		√	
Rodriguez C, 2007[42]	2007	USA	69991	M	50-74	Prostatic Cancer	9	Event/Total	√	√			
N'Kontchou G, 2006[43]	2006	France	771	M/F	61.4 (mean)	Hepatocellular Carcinoma	8	Event/Total	√	√			
Niwa Y, 2005[44]	2005	Japan	36456	F	40-79	Ovarian Cancer	8	Event/Total	√	√		√	
Patel AV, 2005[45]	2005	USA	145627	M/F	50-74	Pancreatic Cancer	8	Event/Total	√	√			
Kuriyama S, 2005[46]	2005	Japan	27539	M/F	≥40	Esophageal Cancer, Gastric Cancer, Colorectal Cancer, Liver Cancer, Gallbladder Cancer, Pancreatic Cancer, Lung Cancer, Kidney Cancer, Breast Cancer, Endometrial Cancer, Cervical Cancer, Ovarian Cancer	8	Event/Total	√	√		√	√
Lin J, 2004[47]	2004	USA	37671	M/F	≥45	Breast Cancer	8	Event/Total	√	√			
Moore LL, 2004[48]	2004	USA	7566	M/F	30-79	Colon Cancer	9	Event/Total	√	√		√	√
Park B, 2022[49]	2022	South Korea	184931	F	≥40	Breast Cancer	9	Event/Total			√		
Kim SJ, 2021[50]	2021	Canada	3853	F	18	Ovarian Cancer	7	Event/Total			√		
da Silva M, 2018[51]	2018	Norway	80930	M/F	30-70	Breast, Colon, Rectal, Ovarian, Pancreatic, Kidney, Endometrial Cancer	9	Event/Total			√		

Chadid S, 2018[52]	2018	USA	3850	M/F	≥60	Female reproductive (postmenopausal, Breast, Uterine/Endometrial and Ovarian), Colon, Rectum, Stomach, Liver, Gallbladder, Pancreas, Kidney, Thyroid, Esophageal Adenocarcinoma, Leukaemia， Non Hodgkin Lymphoma and Multiple Myeloma	7	Event/Total			√	
Kim SJ,2018[53]	2018	Canada & Poland	3493	F	18	Breast Cancer	7	Event/Total			√	
Rosner B, 2017[54]	2017	USA	74177	F	30-55	Breast Cancer	7	Event/Total			√	
Alsaker MD, 2013[55]	2013	Norway	28153	F	≥20	Breast Cancer	9	Event/Total			√	
Chamberlain C, 2011[56]	2011	United Kingdom	20991	M	≥20	Prostatic Cancer	9	Event/Total			√	
Bassett JK, 2010[57]	2010	Australia	39626	M/F	40-69	Colon Cancer	9	Event/Total			√	
Thygesen LC, 2008[58]	2008	USA	46349	F	40-75	Colon Cancer(male)	8	Event/Total			√	
Klintman M, 2022[59]	2022	Sweden	35412	F	62（median）	Breast Cancer	7	Event/Total	√	√		√
Shao F, 2022[60]	2022	the People's Republic of China	450482	M/F	40-69	Lung Cancer	8	Event/Total	√	√		
Song H, 2022[61]	2022	Korea	40432	F	≥40	Breast Cancer	7	Event/Total	√	√		√
Urbute A, 2022[62]	2022	Denmark	461646	F	≤49	Ovarian Cancer	7	Event/Total	√	√		√
Park B, 2022 [63]	2022	Korea	6097686	F	≥40	Endometrial Cancer	7	Event/Total	√	√		√

Yang W, 2022[64]	2022	the People's Republic of China	138 922	M/F	20-50	Liver Cancer, Biliary Tract Cancer	8	Event/Total	√	√		√
Choi IY, 2021[65]	2021	Korea	6272367	M/F	≥40	Gastric Cancer	9	Event/Total	√	√		√
Shin CM, 2017[66]	2017	Korea	408931	M/F	≥20	Colorectal Cancer	7	Event/Total	√	√		√

Abbreviation: M, male; F, female; NA, not available; HR, hazard ratio.

1. Pasqual E, O'Brien K, Rinaldi S, Sandler DP, Kitahara CM. Obesity, obesity-related metabolic conditions, and risk of thyroid cancer in women: results from a prospective cohort study (Sister Study). *The Lancet Regional Health - Americas*. 2023;23doi:10.1016/j.lana.2023.100537
2. Safizadeh F, Mandic M, Pulte D, Niedermaier T, Hoffmeister M, Brenner H. The underestimated impact of excess body weight on colorectal cancer risk: Evidence from the UK Biobank cohort. *British Journal of Cancer*. 2023;doi:10.1038/s41416-023-02351-6
3. Nguyen DN, Kim JH, Kim MK. Association of Metabolic Health and Central Obesity with the Risk of Thyroid Cancer: Data from the Korean Genome and Epidemiology Study. *Cancer Epidemiol Biomarkers Prev*. Mar 1 2022;31(3):543-553. doi:10.1158/1055-9965.EPI-21-0255
4. Lee HW, Huang D, Shin WK, et al. Obesity at early adulthood increases risk of gastric cancer from the Health Examinees-Gem (HEXA-G) study. *PLoS One*. 2022;17(2):e0260826. doi:10.1371/journal.pone.0260826
5. Mao D, Lau ESH, Wu H, et al. Risk Associations of Glycemic Burden and Obesity With Liver Cancer-A 10-Year Analysis of 15,280 Patients With Type 2 Diabetes. *Hepatol Commun*. Jun 2022;6(6):1350-1360. doi:10.1002/hep4.1891
6. Miyata H, Shirai K, Muraki I, Iso H, Tamakoshi A. Associations of Body Mass Index, Weight Change, Physical Activity, and Sedentary Behavior With Endometrial Cancer Risk Among Japanese Women: The Japan Collaborative Cohort Study. *J Epidemiol*. Dec 5 2021;31(12):621-627. doi:10.2188/jea.JE20200145
7. Abe SK, Narita S, Saito E, et al. Body Mass Index, Height, Weight Change, and Subsequent Lung Cancer Risk: The Japan Public Health Center-Based Prospective Study. *Cancer Epidemiol Biomarkers Prev*. Sep 2021;30(9):1708-1716. doi:10.1158/1055-9965.EPI-21-0195
8. Smith SG, Sestak I, Morris MA, et al. The impact of body mass index on breast cancer incidence among women at increased risk: an observational study from the International Breast Intervention Studies. *Breast Cancer Res Treat*. Jul 2021;188(1):215-223. doi:10.1007/s10549-021-06141-7
9. Baumeister SE, Schlecht I, Trabert B, Nolde M, Meisinger C, Leitzmann MF. Anthropometric risk factors for ovarian cancer in the NIH-AARP Diet and Health Study. *Cancer Causes Control*. Mar 2021;32(3):231-239. doi:10.1007/s10552-020-01377-y
10. Maliniak ML, Gapstur SM, McCullough LE, et al. Joint associations of physical activity and body mass index with the risk of established excess body

fatness-related cancers among postmenopausal women. *Cancer Causes Control*. Feb 2021;32(2):127-138. doi:10.1007/s10552-020-01365-2
11. Sanikini H, Muller DC, Chadeau-Hyam M, Murphy N, Gunter MJ, Cross AJ. Anthropometry, body fat composition and reproductive factors and risk of oesophageal and gastric cancer by subtype and subsite in the UK Biobank cohort. *PLoS One*. 2020;15(10):e0240413. doi:10.1371/journal.pone.0240413
12. Luo J, Chen X, Manson JE, et al. Birth weight, weight over the adult life course and risk of breast cancer. *Int J Cancer*. Jul 1 2020;147(1):65-75. doi:10.1002/ijc.32710
13. Minami CA, Zabor EC, Gilbert E, et al. Do Body Mass Index and Breast Density Impact Cancer Risk Among Women with Lobular Carcinoma In Situ? *Ann Surg Oncol*. Jun 2020;27(6):1844-1851. doi:10.1245/s10434-019-08126-9
14. Renehan AG, Pegington M, Harvie MN, et al. Young adulthood body mass index, adult weight gain and breast cancer risk: the PROCAS Study (United Kingdom). *Br J Cancer*. May 2020;122(10):1552-1561. doi:10.1038/s41416-020-0807-9
15. Sanikini H, Muller DC, Sophiea M, et al. Anthropometric and reproductive factors and risk of esophageal and gastric cancer by subtype and subsite: Results from the European Prospective Investigation into Cancer and Nutrition (EPIC) cohort. *Int J Cancer*. Feb 15 2020;146(4):929-942. doi:10.1002/ijc.32386
16. Everatt R, Virviciute D, Tamosiunas A. Body mass index and other risk factors for kidney cancer in men: a cohort study in Lithuania. *Cent Eur J Public Health*. Dec 2019;27(4):272-278. doi:10.21101/cejph.a5080
17. Hirabayashi M, Inoue M, Sawada N, et al. Effect of body-mass index on the risk of gastric cancer: A population-based cohort study in A Japanese population. *Cancer Epidemiol*. Dec 2019;63:101622. doi:10.1016/j.canep.2019.101622
18. Kawachi A, Shimazu T, Budhathoki S, et al. Association of BMI and height with the risk of endometrial cancer, overall and by histological subtype: a population-based prospective cohort study in Japan. *Eur J Cancer Prev*. May 2019;28(3):196-202. doi:10.1097/CEJ.0000000000000449
19. Wakamatsu M, Sugawara Y, Zhang S, Tanji F, Tomata Y, Tsuji I. Weight change since age 20 and incident risk of obesity-related cancer in Japan: a pooled analysis of the Miyagi Cohort Study and the Ohsaki Cohort Study. *Int J Cancer*. Mar 1 2019;144(5):967-980. doi:10.1002/ijc.31743
20. Zohar L, Rottenberg Y, Twig G, et al. Adolescent overweight and obesity and the risk for pancreatic cancer among men and women: a nationwide study of 1.79 million Israeli adolescents. *Cancer*. Jan 1 2019;125(1):118-126. doi:10.1002/cncr.31764
21. Schoemaker MJ, Nichols HB, Wright LB, et al. Association of Body Mass Index and Age With Subsequent Breast Cancer Risk in Premenopausal Women. *JAMA Oncol*. Nov 1 2018;4(11):e181771. doi:10.1001/jamaoncol.2018.1771
22. Xu HL, Zhang ML, Yan YJ, et al. Body mass index and cancer risk among Chinese patients with type 2 diabetes mellitus. *BMC Cancer*. Aug 6 2018;18(1):795. doi:10.1186/s12885-018-4675-0
23. Dickerman BA, Ahearn TU, Giovannucci E, et al. Weight change, obesity and risk of prostate cancer progression among men with clinically localized prostate cancer. *Int J Cancer*. Sep 1 2017;141(5):933-944. doi:10.1002/ijc.30803

24. Leiba A, Duek A, Afek A, Derazne E, Leiba M. Obesity and related risk of myeloproliferative neoplasms among israeli adolescents. *Obesity (Silver Spring)*. Jul 2017;25(7):1187-1190. doi:10.1002/oby.21863
25. Yamamoto-Honda R, Takahashi Y, Yoshida Y, et al. Body mass index and the risk of cancer incidence in patients with type 2 diabetes in Japan: Results from the National Center Diabetes Database. *J Diabetes Investig*. Nov 2016;7(6):908-914. doi:10.1111/jdi.12522
26. Sponholtz TR, Palmer JR, Rosenberg L, Hatch EE, Adams-Campbell LL, Wise LA. Body Size, Metabolic Factors, and Risk of Endometrial Cancer in Black Women. *Am J Epidemiol*. Feb 15 2016;183(4):259-68. doi:10.1093/aje/kwv186
27. White AJ, Nichols HB, Bradshaw PT, Sandler DP. Overall and central adiposity and breast cancer risk in the Sister Study. *Cancer*. Oct 15 2015;121(20):3700-8. doi:10.1002/cncr.29552
28. Moller H, Roswall N, Van Hemelrijck M, et al. Prostate cancer incidence, clinical stage and survival in relation to obesity: a prospective cohort study in Denmark. *Int J Cancer*. Apr 15 2015;136(8):1940-7. doi:10.1002/ijc.29238
29. de Mutsert R, Sun Q, Willett WC, Hu FB, van Dam RM. Overweight in early adulthood, adult weight change, and risk of type 2 diabetes, cardiovascular diseases, and certain cancers in men: a cohort study. *Am J Epidemiol*. Jun 1 2014;179(11):1353-65. doi:10.1093/aje/kwu052
30. Lam TK, Moore SC, Brinton LA, et al. Anthropometric measures and physical activity and the risk of lung cancer in never-smokers: a prospective cohort study. *PLoS One*. 2013;8(8):e70672. doi:10.1371/journal.pone.0070672
31. Patel AV, Diver WR, Teras LR, Birmann BM, Gapstur SM. Body mass index, height and risk of lymphoid neoplasms in a large United States cohort. *Leuk Lymphoma*. Jun 2013;54(6):1221-7. doi:10.3109/10428194.2012.742523
32. Renehan AG, Flood A, Adams KF, et al. Body mass index at different adult ages, weight change, and colorectal cancer risk in the National Institutes of Health-AARP Cohort. *Am J Epidemiol*. Dec 15 2012;176(12):1130-40. doi:10.1093/aje/kws192
33. Smith L, Brinton LA, Spitz MR, et al. Body mass index and risk of lung cancer among never, former, and current smokers. *J Natl Cancer Inst*. May 16 2012;104(10):778-89. doi:10.1093/jnci/djs179
34. Cecchini RS, Costantino JP, Cauley JA, et al. Body mass index and the risk for developing invasive breast cancer among high-risk women in NSABP P-1 and STAR breast cancer prevention trials. *Cancer Prev Res (Phila)*. Apr 2012;5(4):583-92. doi:10.1158/1940-6207.CAPR-11-0482
35. Laake I, Thune I, Selmer R, Tretli S, Slattery ML, Veierod MB. A prospective study of body mass index, weight change, and risk of cancer in the proximal and distal colon. *Cancer Epidemiol Biomarkers Prev*. Jun 2010;19(6):1511-22. doi:10.1158/1055-9965.EPI-09-0813
36. Sawada N, Inoue M, Sasazuki S, et al. Body mass index and subsequent risk of kidney cancer: a prospective cohort study in Japan. *Ann Epidemiol*. Jun 2010;20(6):466-72. doi:10.1016/j.annepidem.2010.03.008
37. Mijovic T, How J, Pakdaman M, et al. Body mass index in the evaluation of thyroid cancer risk. *Thyroid*. May 2009;19(5):467-72. doi:10.1089/thy.2008.0386

38. Rod NH, Hansen AM, Nielsen J, Schnohr P, Gronbaek M. Low-risk factor profile, estrogen levels, and breast cancer risk among postmenopausal women. *Int J Cancer*. Apr 15 2009;124(8):1935-40. doi:10.1002/ijc.24136
39. Leitzmann MF, Koebnick C, Danforth KN, et al. Body mass index and risk of ovarian cancer. *Cancer*. Feb 15 2009;115(4):812-22. doi:10.1002/cncr.24086
40. Iwasaki M, Otani T, Inoue M, Sasazuki S, Tsugane S, Japan Public Health Center-Based Prospective Study G. Body size and risk for breast cancer in relation to estrogen and progesterone receptor status in Japan. *Ann Epidemiol*. Apr 2007;17(4):304-12. doi:10.1016/j.annepidem.2006.09.003
41. Nothlings U, Wilkens LR, Murphy SP, Hankin JH, Henderson BE, Kolonel LN. Body mass index and physical activity as risk factors for pancreatic cancer: the Multiethnic Cohort Study. *Cancer Causes Control*. Mar 2007;18(2):165-75. doi:10.1007/s10552-006-0100-0
42. Rodriguez C, Freedland SJ, Deka A, et al. Body mass index, weight change, and risk of prostate cancer in the Cancer Prevention Study II Nutrition Cohort. *Cancer Epidemiol Biomarkers Prev*. Jan 2007;16(1):63-9. doi:10.1158/1055-9965.EPI-06-0754
43. N'Kontchou G, Paries J, Htar MT, et al. Risk factors for hepatocellular carcinoma in patients with alcoholic or viral C cirrhosis. *Clin Gastroenterol Hepatol*. Aug 2006;4(8):1062-8. doi:10.1016/j.cgh.2006.05.013
44. Niwa Y, Yatsuya H, Tamakoshi K, et al. Relationship between body mass index and the risk of ovarian cancer in the Japanese population: findings from the Japanese Collaborate Cohort (JACC) study. *J Obstet Gynaecol Res*. Oct 2005;31(5):452-8. doi:10.1111/j.1447-0756.2005.00319.x
45. Patel AV, Rodriguez C, Bernstein L, Chao A, Thun MJ, Calle EE. Obesity, recreational physical activity, and risk of pancreatic cancer in a large U.S. Cohort. *Cancer Epidemiol Biomarkers Prev*. Feb 2005;14(2):459-66. doi:10.1158/1055-9965.EPI-04-0583
46. Kuriyama S, Tsubono Y, Hozawa A, et al. Obesity and risk of cancer in Japan. *Int J Cancer*. Jan 1 2005;113(1):148-57. doi:10.1002/ijc.20529
47. Lin J, Zhang SM, Cook NR, Rexrode KM, Lee IM, Buring JE. Body mass index and risk of colorectal cancer in women (United States). *Cancer Causes Control*. Aug 2004;15(6):581-9. doi:10.1023/B:CACO.0000036168.23351.f1
48. Moore LL, Bradlee ML, Singer MR, et al. BMI and waist circumference as predictors of lifetime colon cancer risk in Framingham Study adults. *Int J Obes Relat Metab Disord*. Apr 2004;28(4):559-67. doi:10.1038/sj.ijo.0802606
49. Park B. Changes in weight and waist circumference during menopausal transition and postmenopausal breast cancer risk. *Int J Cancer*. May 1 2022;150(9):1431-1438. doi:10.1002/ijc.33906
50. Kim SJ, Lubinski J, Huzarski T, et al. Weight Gain and the Risk of Ovarian Cancer in BRCA1 and BRCA2 Mutation Carriers. *Cancer Epidemiol Biomarkers Prev*. Nov 2021;30(11):2038-2043. doi:10.1158/1055-9965.EPI-21-0296
51. da Silva M, Weiderpass E, Licaj I, Lissner L, Rylander C. Excess body weight, weight gain and obesity-related cancer risk in women in Norway: the Norwegian Women and Cancer study. *Br J Cancer*. Aug 2018;119(5):646-656. doi:10.1038/s41416-018-0240-5
52. Chadid S, Singer MR, Kreger BE, Bradlee ML, Moore LL. Midlife weight gain is a risk factor for obesity-related cancer. *Br J Cancer*. Jun 2018;118(12):1665-1671. doi:10.1038/s41416-018-0106-x

53. Kim SJ, Huzarski T, Gronwald J, et al. Prospective evaluation of body size and breast cancer risk among BRCA1 and BRCA2 mutation carriers. *Int J Epidemiol*. Jun 1 2018;47(3):987-997. doi:10.1093/ije/dyy039

54. Rosner B, Eliassen AH, Toriola AT, et al. Weight and weight changes in early adulthood and later breast cancer risk. *Int J Cancer*. May 1 2017;140(9):2003-2014. doi:10.1002/ijc.30627

55. Alsaker MD, Janszky I, Opdahl S, Vatten LJ, Romundstad PR. Weight change in adulthood and risk of postmenopausal breast cancer: the HUNT study of Norway. *Br J Cancer*. Sep 3 2013;109(5):1310-7. doi:10.1038/bjc.2013.403

56. Chamberlain C, Romundstad P, Vatten L, Gunnell D, Martin RM. The association of weight gain during adulthood with prostate cancer incidence and survival: a population-based cohort. *Int J Cancer*. Sep 1 2011;129(5):1199-206. doi:10.1002/ijc.25739

57. Bassett JK, Severi G, English DR, et al. Body size, weight change, and risk of colon cancer. *Cancer Epidemiol Biomarkers Prev*. Nov 2010;19(11):2978-86. doi:10.1158/1055-9965.EPI-10-0543

58. Thygesen LC, Gronbaek M, Johansen C, Fuchs CS, Willett WC, Giovannucci E. Prospective weight change and colon cancer risk in male US health professionals. *Int J Cancer*. Sep 1 2008;123(5):1160-5. doi:10.1002/ijc.23612

59. Klintman M, Rosendahl AH, Randeris B, et al. Postmenopausal overweight and breast cancer risk; results from the KARMA cohort. *Breast Cancer Res Treat*. Nov 2022;196(1):185-196. doi:10.1007/s10549-022-06664-7

60. Shao F, Chen Y, Xu H, et al. Metabolic Obesity Phenotypes and Risk of Lung Cancer: A Prospective Cohort Study of 450,482 UK Biobank Participants. *Nutrients*. Aug 17 2022;14(16)doi:10.3390/nu14163370

61. Song H, Jeong A, Tran TXM, Lee J, Kim M, Park B. Association between Micronutrient Intake and Breast Cancer Risk According to Body Mass Index in South Korean Adult Women: A Cohort Study. *Nutrients*. Jun 26 2022;14(13)doi:10.3390/nu14132644

62. Urbute A, Frederiksen K, Kjaer SK. Early adulthood overweight and obesity and risk of premenopausal ovarian cancer, and premenopausal breast cancer including receptor status: prospective cohort study of nearly 500,000 Danish women. *Ann Epidemiol*. Jun 2022;70:61-67. doi:10.1016/j.annepidem.2022.03.013

63. Park B. Associations between obesity, metabolic syndrome, and endometrial cancer risk in East Asian women. *J Gynecol Oncol*. Jul 2022;33(4):e35. doi:10.3802/jgo.2022.33.e35

64. Yang W, Zeng X, Petrick JL, et al. Body mass index trajectories, weight gain and risks of liver and biliary tract cancers. *JNCI Cancer Spectr*. Aug 12 2022;6(4)doi:10.1093/jncics/pkac056

65. Choi IY, Choi YJ, Shin DW, et al. Association between obesity and the risk of gastric cancer in premenopausal and postmenopausal women: A nationwide cohort study. *J Gastroenterol Hepatol*. Oct 2021;36(10):2834-2840. doi:10.1111/jgh.15558

66. Shin CM, Han K, Lee DH, et al. Association Among Obesity, Metabolic Health, and the Risk for Colorectal Cancer in the General Population in Korea

Using the National Health Insurance Service-National Sample Cohort. *Dis Colon Rectum*. Nov 2017;60(11):1192-1200. doi:10.1097/DCR.0000000000000876

Figure S1. The result of the quality of the included studies assessed by the Newcastle-Ottawa Scale

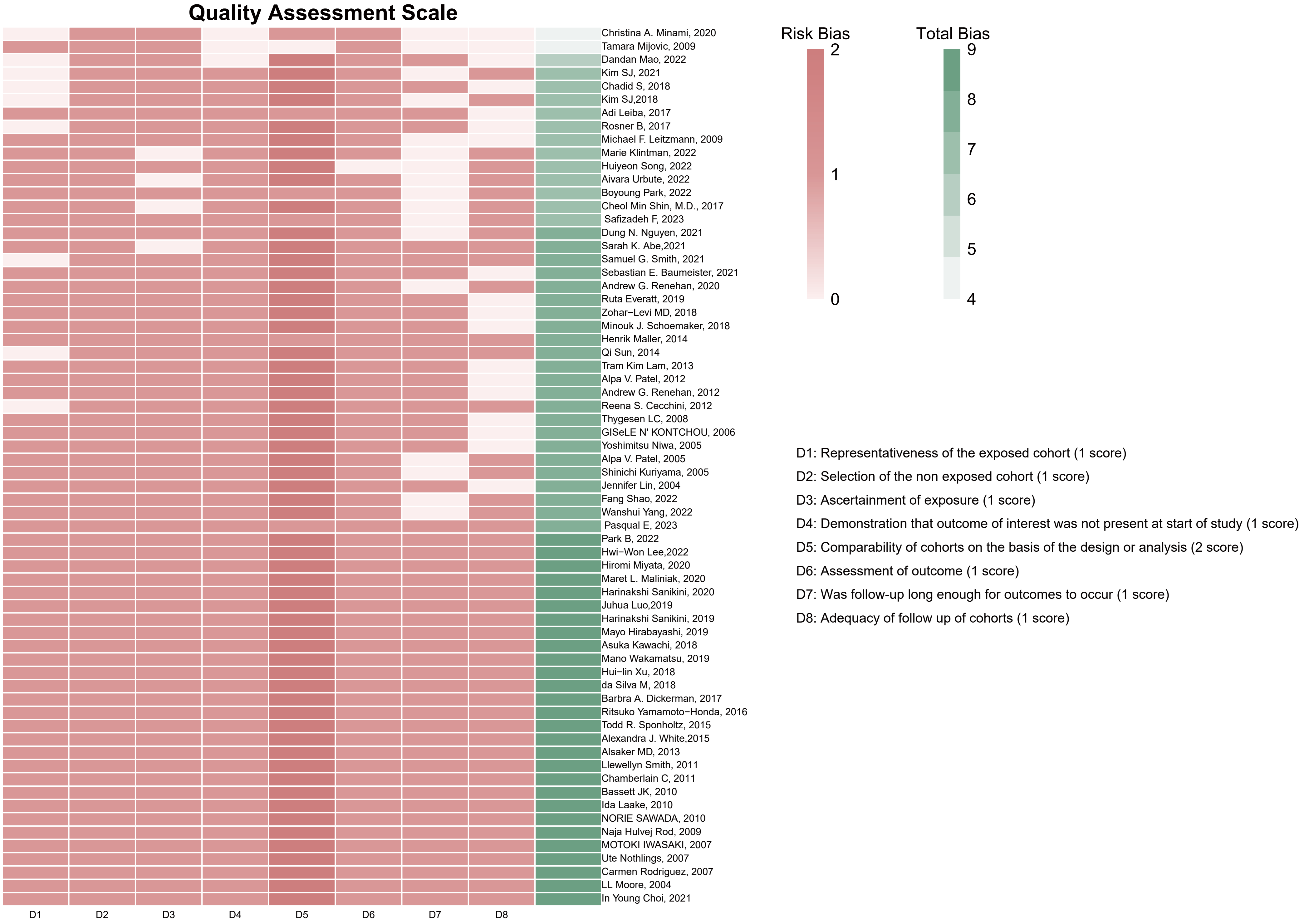

Figure S2: Forest plots of the studies(pooling the HRs) analyzing the risk of overall cancer.

Study or Subgroup	log[Hazard Ratio]	SE	Weight	Hazard Ratio IV, Random, 95% CI
Adi Leiba, 2017 (Obese)	0.7799	0.2641	7.8%	2.18 [1.30, 3.66]
Adi Leiba, 2017 (Overweight)	-0.1356	0.183	13.4%	0.87 [0.61, 1.25]
Christina A. Minami, 2020 (Obese)	0.0063	0.2554	8.3%	1.01 [0.61, 1.66]
Christina A. Minami, 2020 (Overweight)	0.0005	0.175	14.2%	1.00 [0.71, 1.41]
Christina A. Minami, 2020 (Very obese)	0.2248	0.319	5.7%	1.25 [0.67, 2.34]
Sebastian E. Baumeister, 2021 (Obese)	0.1066	0.1025	24.3%	1.11 [0.91, 1.36]
Sebastian E. Baumeister, 2021 (Overweight)	-0.0312	0.0916	26.3%	0.97 [0.81, 1.16]
**Total (95% CI)**			**100.0%**	**1.08 [0.92, 1.27]**

Heterogeneity: Tau² = 0.02; Chi² = 10.17, df = 6 (P = 0.12); I² = 41%
Test for overall effect: Z = 0.90 (P = 0.37)

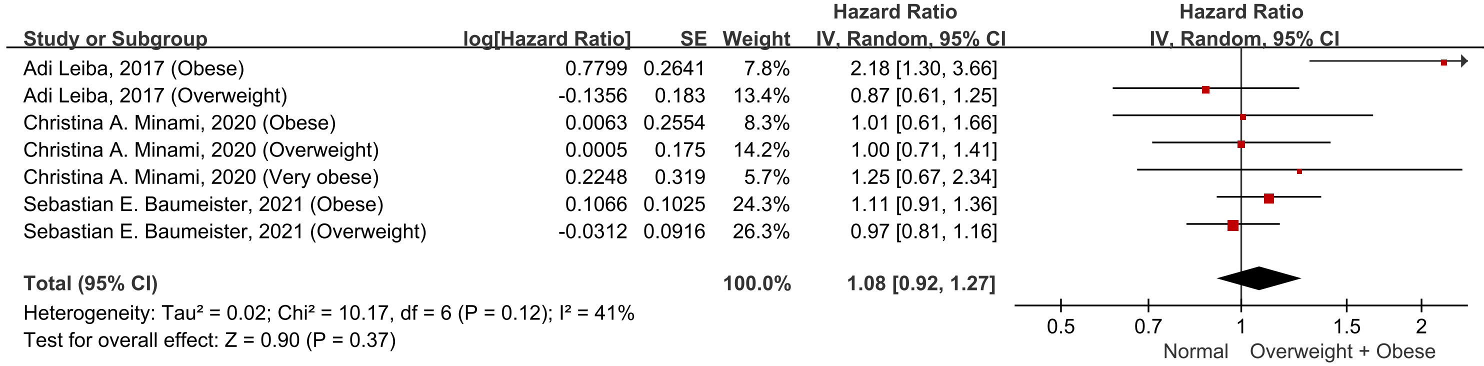

Heterogeneity: Tau² = 0.02; Chi² = 10.17, df = 6 (P = 0.12); I² = 41%
Test for overall effect: Z = 0.90 (P = 0.37)

log[Hazard Ratio]	SE	Weight	Hazard Ratio IV, Fixed, 95% CI
0.7799	0.2641	4.5%	2.18 [1.30, 3.66]
-0.1356	0.183	9.4%	0.87 [0.61, 1.25]
0.0063	0.2554	4.8%	1.01 [0.61, 1.66]
0.0005	0.175	10.3%	1.00 [0.71, 1.41]
0.2248	0.319	3.1%	1.25 [0.67, 2.34]
0.1066	0.1025	30.1%	1.11 [0.91, 1.36]
-0.0312	0.0916	37.7%	0.97 [0.81, 1.16]
		**100.0%**	**1.05 [0.94, 1.17]**

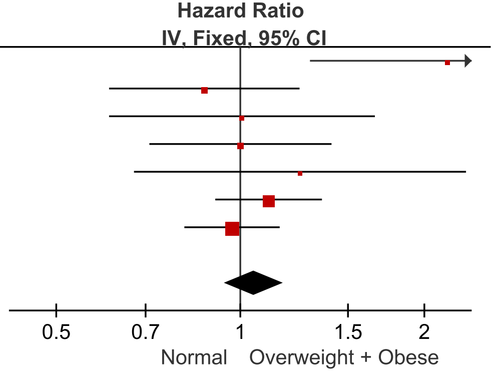

Heterogeneity: Chi² = 10.17, df = 6 (P = 0.12); I² = 41%
Test for overall effect: Z = 0.89 (P = 0.37)

Figure S3. Summary risk estimated by different BMI comparisons in Panel A. digestive system cancer and Panel B.female reproductive system cancer. Abbreviations: RR, relative risk; CI, confidence interval.

A

Comparison Groups	No. of Studies	Expriment		Control		Random Effects Model		Fixed Effects Model		I²
		Event	Total	Event	Total	RR(95%CI)	P	RR(95%CI)	P	
Underweight vs Normal	7	1230	286674	29427	5995457	1.16 [0.92, 1.46]	0.2	1.11 [1.05, 1.18]	0.0003	61%
Overweight vs Normal	13	20054	2783761	31652	6343844	1.22 [1.13, 1.31]	<0.0001	1.20 [1.18, 1.22]	<0.0001	83%
Obesity vs Normal	13	5300	733462	31652	6343844	1.39 [1.24, 1.55]	<0.0001	1.28 [1.24, 1.32]	<0.0001	87%
Overweight or Obesity vs Normal	13	25354	3517313	31652	6343844	1.27 [1.17, 1.37]	<0.0001	1.21 [1.19, 1.23]	<0.0001	89%

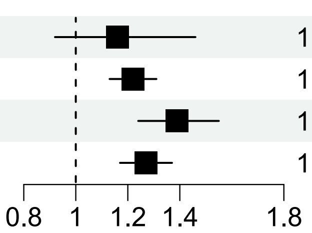

B

Comparison Groups	No. of Studies	Expriment		Control		Random Effects Model		Fixed Effects Model		I²
		Event	Total	Event	Total	RR(95%CI)	P	RR(95%CI)	P	
Underweight vs Normal	6	71	8299	3134	113409	0.71 [0.56, 0.89]	0.004	0.70 [0.55, 0.88]	0.002	0%
Overweight vs Normal	8	3882	174701	4686	431999	1.12 [1.00, 1.25]	0.05	1.10 [1.05, 1.14]	<0.0001	76%
Obesity vs Normal	8	2797	99285	4686	431999	1.29 [1.03, 1.62]	0.03	1.21 [1.15, 1.26]	<0.0001	93%
Overweight or Obesity vs Normal	8	6679	273986	4686	431999	1.18 [1.02, 1.36]	0.02	1.14 [1.10, 1.19]	<0.0001	90%

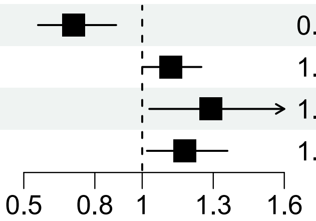

Figure S4. Summary risk of colorectal cancer estimated by different BMI comparisons in men and women.
Abbreviations: RR, relative risk; CI, confidence interval.

Comparison Groups and Sex	No. of Studies	Expriment		Control		I²	Random Effects Model		Subgroup Differences		Fixed Effects Model		Subgroup Differences	
		Event	Total	Event	Total		RR(95%CI)	P	I²	P	RR(95%CI)	P	I²	P
Underweight or Normal vs Overweight or Obesity														
Men	5	2470	141626	1127	83134	68%	1.30 [1.06, 1.59]	0.01	0%	0.63	1.21 [1.13, 1.30]	<0.0001	0%	0.37
Women	5	1068	78084	883	84453	81%	1.41 [1.08, 1.86]	0.01			1.28 [1.17, 1.40]	<0.0001		
Overweight vs Normal														
Men	4	1615	121415	872	81452	46%	1.30 [1.11, 1.51]	0.0008	0%	0.79	1.25 [1.15, 1.36]	<0.0001	0%	0.74
Women	4	576	49815	744	82735	62%	1.34 [1.09, 1.64]	0.005			1.28 [1.15, 1.43]	<0.0001		
Obesity vs Normal														
Men	4	322	19612	872	81452	73%	1.68 [1.19, 2.39]	0.003	0%	0.85	1.50 [1.32, 1.70]	<0.0001	0%	0.35
Women	4	328	27976	744	82735	89%	1.78 [1.10, 2.87]	0.02			1.37 [1.21, 1.56]	<0.0001		
Overweight or Obesity vs Normal														
Men	4	1937	141027	872	81452	62%	1.35 [1.13, 1.61]	0.001	0%	0.72	1.29 [1.19, 1.40]	<0.0001	0%	0.69
Women	4	2841	218818	1616	164187	83%	1.43 [1.09, 1.88]	0.009			1.33 [1.20, 1.46]	<0.0001		

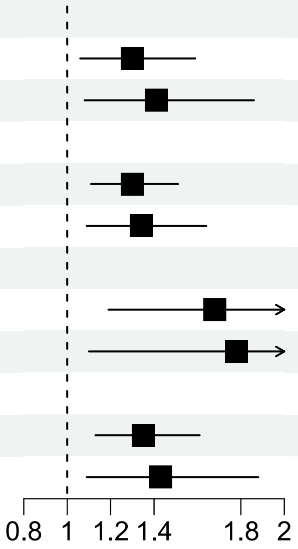

Figure S5. Funnel Plots. Panel A. Overweight vs. Normal Weight; Panel B. Overweight or Obesity vs. Normal Weight; Panel C.Overweight or Obesity vs. Underweight or Normal Weight; Panel D.Obesity vs. Normal Weight; Panel E. Weight Gain vs. Weight Loss; Panel F. Weight Gain vs. Weight Loss; Panel F. Underweight vs. Normal Weight.

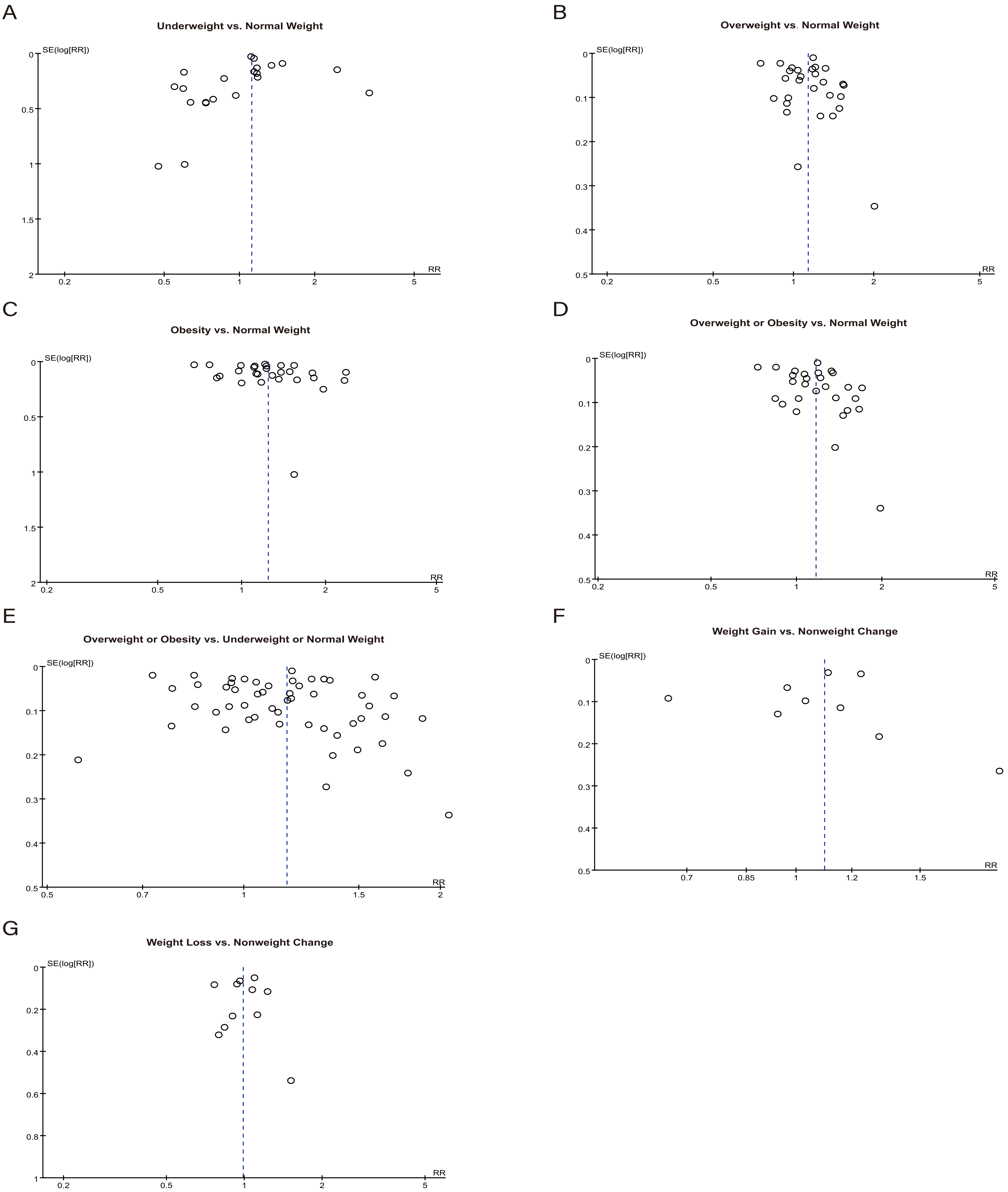

Supplement: Online Supplementary Document. [file jogh-14-04067-s001.pdf]
